# Unveiling the importance of SIRT5 for cardiac health and disease in an era of increasing longevity

**DOI:** 10.1007/s11357-025-02021-w

**Published:** 2025-11-19

**Authors:** Arkadiusz Grzeczka, Szymon Graczyk, Pawel Kordowitzki

**Affiliations:** 1 Department of Basic and Preclinical Sciences, Faculty of Biological and Veterinary Sciences, Nicolaus Copernicus University, Torun, Poland; 2 Department of Gynecology, European Competence Center for Ovarian Cancer, Charité Medical University, Berlin, Germany

**Keywords:** Sirtuins, Post-translational protein modifications, Succinylation, Cardiovascular diseases, Mitochondrial metabolism

## Abstract

As the world’s population ages, strategies to promote healthy longevity are critical. SIRT5, belonging to the sirtuin family, plays a key role in regulating cellular metabolism and mitochondrial homeostasis by removing post-translational modifications such as succinylation, malonylation, and glutarylisation from lysine residues of proteins. Herein, we discuss the multifunctionality of SIRT5, its impact on heart function, and its involvement as a target in geroscience for the pathogenesis of cardiovascular diseases, including heart failure, diabetic cardiomyopathy, cardiac hypertrophy, and ischemia-reperfusion injury. Research indicates that SIRT5 modulates metabolic pathways, oxidative stress response, and apoptosis, making it a potential therapeutic target for extending the health span and lifespan. However, its role is complex and context-dependent, exhibiting protective and harmful effects in various pathological conditions.

## Introduction

The increasing prominence of aging as the primary risk factor for life-threatening diseases, such as cardiovascular diseases, has significantly propelled the pursuit of longevity. The heart muscle requires a continuous and well-coordinated energy metabolism throughout life. ATP production primarily occurs in the mitochondria, which house a multitude of enzymes involved in key metabolic pathways [1]. These organelles integrate several critical processes, including the tricarboxylic acid (TCA) cycle, fatty acid oxidation, and electron transport along the mitochondrial membrane [2]. The efficiency of these systems relies on the precise regulation of enzyme activity, governed by both genetic and epigenetic mechanisms [3]. Regulation of gene expression for enzymes, transport proteins, and components of the antioxidant defense system is particularly important [3]. Among the most prominent protein modifications are phosphorylation, glycation, and ubiquitination, while additional modifications, such as succinylation, malonylation, glutarylation, and acetylation, further refine and regulate the cellular metabolic landscape [4]. Dynamic changes occurring in the developing heart require precise regulatory mechanisms and metabolic switches to meet the evolving demands of the maturing myocardium [5]. In heart failure, both glycolysis and mitochondrial oxidative metabolism are disrupted due to transcriptional changes in key enzymes of these pathways and imbalances in the NAD⁺/NADH redox state [6].

**Fig. 1:**
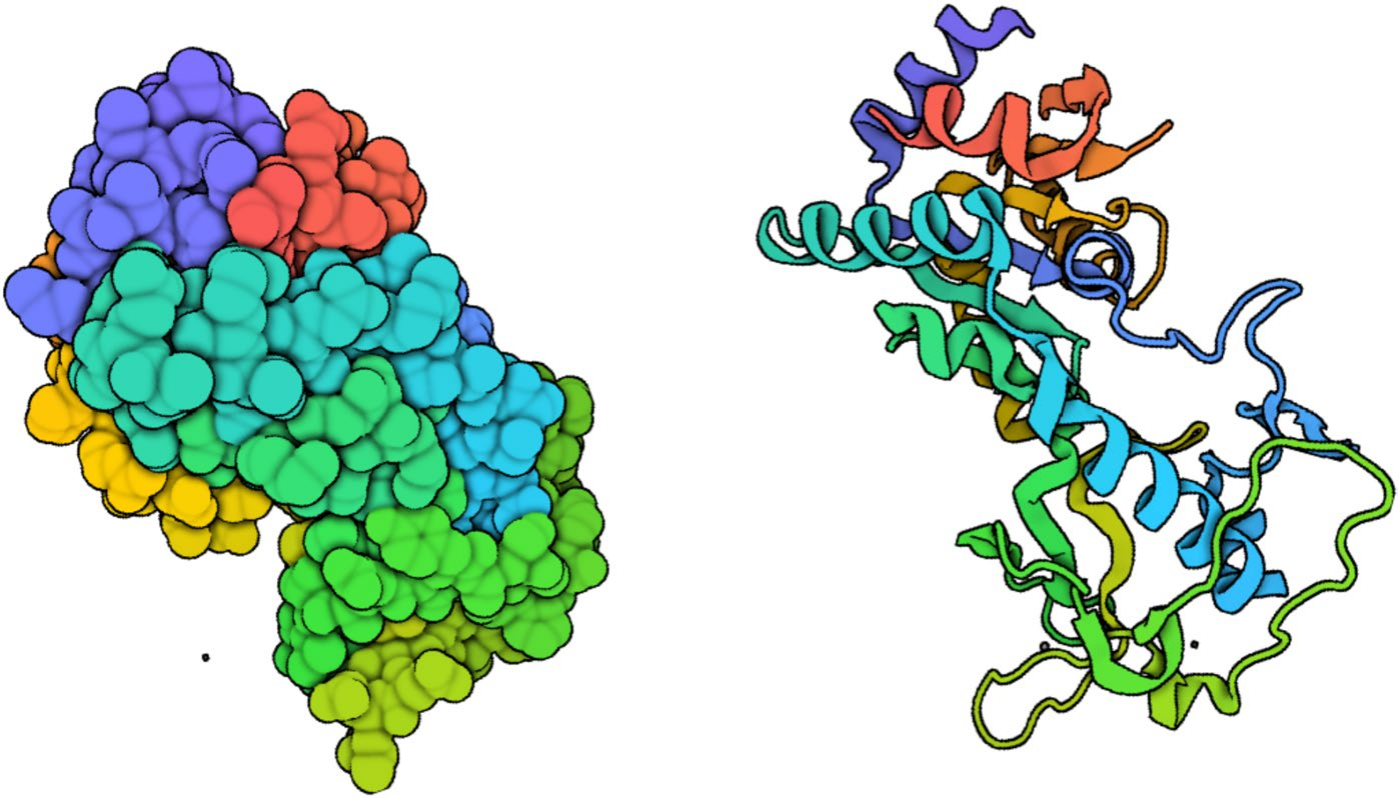
3D protein structure of SIRT 5

Sirtuins, including SIRT5, are NAD⁺-dependent enzymes that play a key role in regulating fundamental cellular processes such as metabolism, DNA repair, and stress responses, and aging [7, 8]. Functionally, sirtuins interact with numerous critical molecular targets; they deacetylate proteins such as PGC-1α (a regulator of mitochondrial biogenesis), p53 (a tumor suppressor), FOXO transcription factors (associated with longevity), NF-κB (involved in the inflammatory response), and MnSOD (a mitochondrial antioxidant enzyme) [7]. Sirtuin 5 (Fig. 1) is distinct among sirtuins due to its ability to remove specific acyl post-translational modifications (PTMs), including succinylation, malonylation, and glutarylation, thereby modulating the function of both mitochondrial and cytoplasmic proteins. In the context of cardiovascular disease and aging, SIRT5 is essential for maintaining energy homeostasis, mitigating oxidative stress, and regulating metabolic pathways [9]. Dysregulation of SIRT5 activity has been linked to various cardiovascular pathologies, including heart failure, diabetic cardiomyopathy, and ischemia-reperfusion injury [10–12].

Herein, the current understanding of the role of SIRT5 in cardiac physiology and disease is emphasized, elucidating its therapeutic potential and the complexity of its molecular mechanisms. This review will also examine the evidence regarding changes in SIRT5 expression during cardiac and vascular aging, elucidating its contributions to age-related cardiovascular decline and its intricate relationships with established aging pathways. Furthermore, this review will discuss the potential of targeting SIRT5 as a geroprotective intervention to mitigate age-associated cardiovascular pathologies.

## Biological framework of SIRT5 activity

Through this mechanism, SIRT5 influences several major biochemical pathways, including the tricarboxylic acid (TCA) cycle, urea cycle, glycolysis, fatty acid β-oxidation, carbohydrate metabolism, and redox balance [13] (Table 1).

**Table 1:** Biological activity of SIRT5

Proces	Target (enzyme/protein)	Type of modification regulated by SIRT5	The effect of SIRT5	References
Role of SIRT5 in control of the urea cycle and nitrogen metabolism				
Ammonia detoxification	Carbamoyl phosphate synthetase 1 (CPS1)	Deacetylation, desuccinylation, deglutarylization	Improved ammonia detoxification; lack of SIRT5 leads to hyperammonemia	[14] [15]
Glutamine metabolism	Glutaminase 2 (GLS2)	Desuccinylation	Reduction in ammonia and glutamate production	[16]
	Mitochondrial proteins	Demethylation and desuccinylation	Stabilization of glutamine metabolism and reduction of ammonia-induced autophagy	[17]
Purine metabolism	Urate oxidase (UOX)	Deacetylation	Activation of UOX; increased purine metabolism in liver mitochondria	[18]
Role of SIRT5 in regulation of energy metabolism				
Glycolysis	GAPDH	Demalonylation	GAPDH activation	[19]
	PKM2	Desuccinylation	PKM2 activation	[20]
Pyruvate conversion	STAT3	Deacetylation	Reduction in PDC activity and ATP synthesis, limits the overproduction of acetyl-CoA	[21] [22]
Tricarboxylic acid (TCA) cycle	IDH2	Desuccinylation	Activation of IDH2 leads to increased production of α-ketoglutarate and NADH	[22] [23]
	HADH	Desuccinylation	Limiting excessive energy metabolism, protection against oxidative stress and mitochondrial dysfunction	[22]
Electron transport chain (ETC)	Complex I, cytochrome c	Desuccinylation	Regulation of mitochondrial ATP production efficiency	[24] [25]
	SDHA	Desuccinylation	Regulation of SDHA activity; SIRT5 deficiency leads to impaired cellular respiration	[24]
	SDHA and SDHB	Desuccinylation	Improves subunit interactions, supports electron transfer, increases enzymatic activity, reduces the risk of lactic acidosis	[25]
Role of SIRT5 in β-oxidation, ketogenesis, and regulation of lipid metabolism				
β-oxidation of fatty acids (FAO)	VLCAD, ECHA	Deacylation (with SIRT3), desuccinylation	Activation of FAO enzymes leads to increased fatty acid oxidation	[26]
Ketogenesis	HMG-CoA synthase 2	Desuccinylation	Activation of HMGCS2 leads to increased production of ketone bodies during starvation	[26]
Role of SIRT5 in stress response				
Oxidative stress	SOD1	Desuccinylation	Increased ROS neutralization capacity	[27]
Antiviral	TBK1	Desuccinylation, demalonylation	Activation of NF-κB and IRF pathways; enhancement of antiviral response	[28]
Apoptosis	p53	Desuccinylation	Maintaining p53 activity	[29]
Proteostasis	GCDH	Deglutarylization	Restoration of GCDH activity, regulation of lysine and tryptophan degradation	[15, 30]

## Sirtuin 5 and aging

Genetic studies indicate that SIRT5 variants may contribute to human longevity, suggesting that this mitochondrial deacylase plays a role in lifespan regulation through its impact on energy metabolism and cellular stress resistance [31]. Mitochondrial dysfunction, a hallmark of metabolic decline during aging, has been linked to decreased NAD+ bioavailability, a critical coenzyme for sirtuin activity [32]. This age-related decline in NAD+ levels, also observed in various cardiovascular pathologies, can consequently impair SIRT5 function and contribute to the progression of age-related cardiac and vascular dysfunction [32]. Specifically, the depletion of mitochondrial sirtuins, including SIRT5, has been demonstrably linked to senescence responses and the secretion of distinct senescence-associated secretory phenotype factors [33]. Moreover, the adequate balance between reactive oxygen species production and antioxidant defense mechanisms, often disrupted during mitochondrial dysfunction, further implicates SIRT5 in maintaining cellular homeostasis crucial for cardiovascular integrity [34, 35]. A thorough understanding of SIRT5’s diverse PTMs and their impact on protein function, particularly within the mitochondrial milieu, is essential for dissecting its contribution to the aging phenotype. The interplay between SIRT5 and other sirtuins, such as SIRT1 and SIRT3, which are also implicated in cardiovascular health and aging, warrants further investigation [36]. SIRT5, primarily localized in the mitochondria, plays a crucial role in regulating metabolic pathways, including fatty acid oxidation and the Krebs cycle, while also influencing cytosolic glycolysis [37]. SIRT5 knockout mice, for instance, exhibit hypersuccinylation in the heart, leading to diminished fatty acid oxidation and reduced ATP production, thereby underscoring its direct involvement in cardiac energy metabolism [38]. This heightened succinylation can consequently impair cardiac function, particularly under metabolic stress conditions, given the heart’s high succinyl CoA concentration [39]. Furthermore, genetic variations or age-related declines in SIRT5 expression or activity could exacerbate these metabolic dysfunctions, contributing to the pathogenesis of age-related cardiovascular diseases. For example, a specific single-nucleotide polymorphism in SIRT5 that increases its protein abundance has been linked to mitochondrial dysfunction, potentially contributing to conditions such as Parkinson’s disease and other age-related pathologies through altered gene activation profiles [40]. In addition, SIRT5 has been implicated in regulating the mitochondrial redox environment, influencing oxidative stress responses crucial for cardiac health [41, 42]. The impact of SIRT5 extends to detoxifying ammonia through its desuccinylation of carbamoyl phosphate synthetase 1, thereby regulating the urea cycle and preventing elevated blood ammonia levels during prolonged fasting [43]. This broad metabolic regulatory capacity positions SIRT5 as a crucial enzyme for maintaining cellular homeostasis, particularly in metabolically active organs like the heart, where its dysregulation could significantly impact energetic efficiency and stress resilience. Indeed, SIRT5, while a weak deacetylase, primarily functions as a potent desuccinylase, demalonylase, and deglutarylase, underscoring its unique enzymatic profile in regulating various PTMs integral to metabolic regulation [40, 44]. These activities are critical for fine-tuning the function of numerous enzymes involved in glycolysis, fatty acid oxidation, the urea cycle, and the tricarboxylic acid cycle, thereby directly impacting cellular bioenergetics [45]. For instance, SIRT5’s desuccinylation of key metabolic enzymes such as carbamoyl phosphate synthetase 1, succinate dehydrogenase, and isocitrate dehydrogenase 2 directly modulates their activity, influencing both ammonia detoxification and mitochondrial energy production [46, 47]. Moreover, dysregulation of SIRT5’s deacylation activity, particularly increased succinylation, has been linked to various cardiovascular diseases, including hypertrophic cardiomyopathy and heart failure, by promoting myocardial fibrosis and reducing cardiac function [47] (Table 2). This highlights the potential for therapeutic interventions targeting SIRT5 to ameliorate cardiac dysfunction and improve cardiovascular outcomes in aging populations [51–53]. Understanding the precise mechanisms by which SIRT5 dysfunction contributes to age-related cardiovascular pathologies is crucial for developing targeted therapeutic strategies [46]. A more detailed description of SIRT5’s relevance for metabolism will be provided in the next section.

**Table 2:** SIRT5 in the aging heart

	Aging process or age-related disease	Mechanism of action of SIRT5	Effect/biological significance	Reference
Blood vessels	Vascular and endothelial aging	Increased SIRT5 expression in the aorta with age; overexpression inhibits the eNOS pathway and impairs vascular relaxation	Impaired endothelial function, vascular wall fibrosis; SIRT5 inhibition improves endothelial function	[48]
Heart	Myocardial fibrosis	Reduced SIRT5 expression leads to increased IDH2 succinylation; SIRT5-promoted IDH2 desuccinylation restores mitochondrial function	Improvement of mitochondrial homeostasis, reduction of oxidative stress and inflammation in cardiomyocytes, inhibition of fibrosis, and improvement of heart function; effect dependent on SIRT5	[49]
Heart	Radiation-induced cardiac aging (RIHD)	SIRT5 regulates deacetylation of mitochondrial Atp5f1c K55	Restores ATP enzymatic activity, prevents cardiomyocyte aging; potential therapeutic target in RIHD	[50]

## Sirtuin 5—a multifunctional regulator of metabolism and epigenetics

**Fig. 2:**
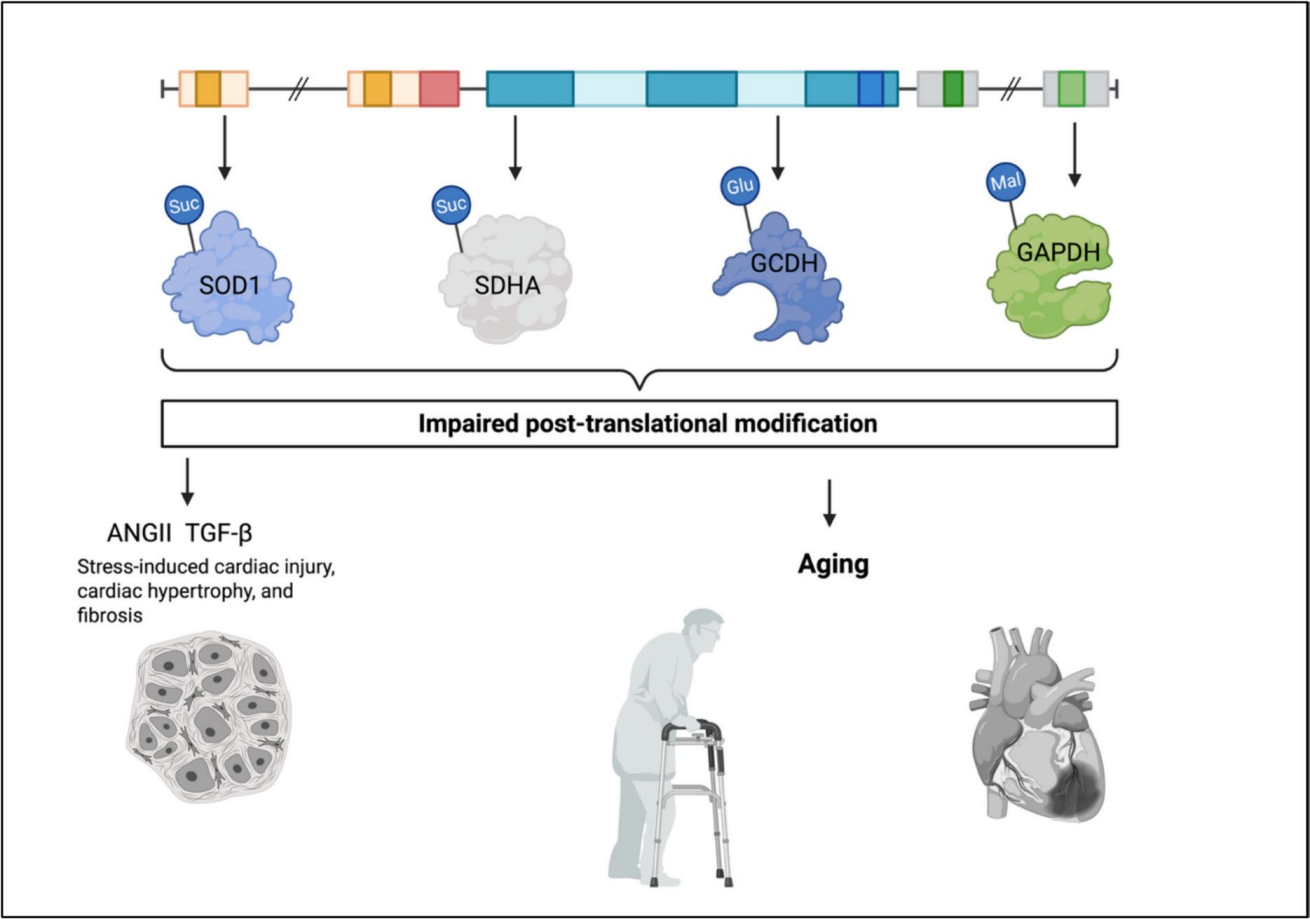
Diagram showing the consequences of a set of incorrectly performed post-translational modifications in the SIRT5 compound. SOD1, superoxide dismutase 1; SDHA, succinate dehydrogenase complex, subunit A; GCDH, glutaryl-CoA dehydrogenase; GAPDH, glyceraldehyde-3-phosphate dehydrogenase. Abnormal PTMs can result in, among other things, abnormal antioxidant defense (SOD1), reduced ATP production (SDHA, GCDH), and abnormal carbohydrate metabolism (GAPDH)

Different protein modifications often overlap in their activity; for example, the global level of succinylation, a modification of lysine residues, can be regulated by other modifications of the same residue, such as acetylation. RNA modifications, such as pseudouridylation (w), 5-methylcytosine (m5C), N6-methyladenosine (m6A), or N4-acetylcytidine (ac4C), can also take place with regard to cardiac fibrosis (Fig. 2) [54]. SIRT5, as a molecule with diverse enzymatic activities, including deacetylation, desuccinylation (Ksu), deglutarylation (Kgl), and demalonylation (Kma), may play a crucial role in maintaining cellular homeostasis and overall health. What distinguishes SIRT5 from other sirtuins is the unusual resistance of its deacetylase activity to nicotinamide inhibition, contrasted by a pronounced sensitivity of its desuccinylase activity to the same inhibitor [55].

### Desuccinylation

Lysine succinylation is an increasingly recognized PTM that has gained status in recent years as a key regulator of protein function in cells. This dynamic process is especially critical in mitochondria [23]. Early research demonstrated that succinylation modifies thousands of sites on hundreds of proteins in mitochondria and other parts of the cell, indicating a broad spectrum of action [22]. Acting as a regulator of succinylation, SIRT5 eliminates succinyl groups from lysines to keep proteins in their active, functional form. This mechanism is of fundamental importance because it allows the regulation of the activity of many important metabolic enzymes, such as pyruvate dehydrogenase and succinate dehydrogenase, which direct energy to the appropriate metabolic pathways [56]. Furthermore, SIRT5 also modulates the antioxidant enzyme SOD1, increasing its ability to neutralize harmful reactive oxygen species. This aspect is particularly important in cancer cells, where the balance of oxidative stress influences their growth and survival [27]. By modulating trophoblast cell survival, SIRT5 contributes to the preservation of cellular equilibrium and offers protection against pregnancy-related hypertensive conditions [57], and prevents neuroinflammatory processes during aging through control of succinylation in microglia [58]. Moreover, SIRT5 activity is fundamental for regulating the inflammatory response, as it desuccinylates the kinase TBK1 in macrophages, restoring its ability to bind IRF3 and TRAF2 and to activate NF-κB and IRF signaling pathways during immune activation [59].

Further reports expanded the picture of the role of SIRT5, showing that this enzyme is a specific regulator of mitochondrial metabolism, controlling, among other things, β-oxidation of fatty acids and ketogenesis [26]. Properly balanced succinylation is therefore essential for the cell’s effective use of energy fuel. In turn, Gibson et al. (2015) indicated that the α-ketoglutarate dehydrogenase complex acts as an enzyme that transfers succinyl groups to other proteins, introducing another layer of regulation of mitochondrial function [60]. The importance of desuccinylation by SIRT5 is also evident in the respiratory chain, particularly in complexes I and II, which are responsible for energy production in mitochondria [24]. The absence of SIRT5 results in reduced activity of these complexes and a decrease in ATP synthesis, which may have far-reaching consequences for the health of the entire organism [25]. Brown adipose tissue, responsible for thermogenesis and glucose regulation, is susceptible to changes in succinylation. SIRT5 regulates the stability and function of UCP1; succinylation of this protein impairs mitochondrial respiration and leads to a loss of metabolic flexibility [61].

Notably, succinylation extends beyond mitochondria. Recent work reveals that CPT1A, an enzyme canonically involved in fatty acid transport, also functions as a succinyltransferase, autonomously modulating cell metabolism and proliferation under glutamine-deficient conditions [62]. This highlights the complexity and multidimensionality of metabolic regulation*.* However, excess succinyl-CoA, resulting from enzymatic disorders such as SUCLA2 ligase deficiency, leads to pathological, global hypersuccinylation. In animal models, increased SIRT5 activity has been shown to reduce this excessive modification, improving survival and suggesting that succinylation imbalances may contribute to the development of severe mitochondrial diseases [45]. Succinylation also affects protein function beyond metabolism. An example is the enzyme hRIDA deaminase, whose succinylation reduces activity and inhibits cell proliferation, which may have implications in cancer [63]. Furthermore, SIRT5 desuccinylates a key lysine 120 on the p53 protein, a major tumor suppressor, affecting its ability to activate the response to DNA damage. The absence of this regulation weakens the function of p53, as confirmed in a double knockout model [29]. This shows that succinylation is an important mechanism controlling metabolism and the cellular response to stress and damage, with broad implications for health and disease.

### Demalonylation

SIRT5 also modulates cell functions by removing malonyl groups from lysine residues of proteins. Malonylation, recently discovered as a new type of PTM, involves the covalent attachment of a malonyl group (derived from malonyl-CoA) to the ε-amino group of lysine [64]. This results in a change in the charge of the residue from positive to negative, which can strongly influence the structure and function of the protein, similar to phosphorylation or succinylation [64–66]. Malonylation is a process that is widespread among living organisms [66]. SIRT5 regulates the malonylation of hundreds of proteins, particularly affecting the glycolytic pathway, and disruption of demalonylation leads to impaired glucose flux and inactivation of key enzymes such as glyceraldehyde-3-phosphate dehydrogenase [19]. Furthermore, studies have shown that SIRT5 reverses malonyl and succinyl modifications, and its specificity toward these groups stems from the structural features of the catalytic pocket [65, 66]. The involvement of SIRT5 malonyl in translation, amino acid metabolism, and selenocysteine synthesis has also been revealed, suggesting a possible role in oncogenesis [67]. At the same time, it has been confirmed that although malonylation structurally resembles other acid modifications (e.g., glutarylisation), it regulates different groups of proteins, giving SIRT5 a unique position in the control of proteostasis [68]. Demalonylation, together with desuccinylation by SIRT5, stabilizes glutamine metabolism and reduces ammonia-induced autophagy, supporting mitochondrial balance [17], and acting on DDX3 through demalonylation, SIRT5 enhances TBK1–IRF3–dependent signaling, strengthening type I interferon responses against viral infection [28]. At the epigenetic level, demalonylation of histones (e.g., H2B_K5) by SIRT5 affects nucleolar organization and rRNA transcription, linking metabolism to gene expression regulation [69]. Finally, in the context of cancer, SIRT5, through transketolase demalonylation, protects colon cancer cells from DNA damage and promotes their growth, indicating a potential therapeutic target [70].

### Deglutarylation

Lysine glutarylation (Kgl) is a relatively newly discovered PTM involving the covalent attachment of a five-carbon glutaryl group to a lysine residue, which significantly alters the charge and properties of the protein. While glutaryl-CoA can promote spontaneous glutarylation, the process is further controlled enzymatically by SIRT5 [15, 30]. As a deglutarylase, SIRT5 modulates lysine glutarylation across numerous proteins, with a predominant impact on mitochondrial metabolic enzymes [15]. Furthermore, SIRT5 plays a key role in the oxidative degradation of lysine and tryptophan [30]. Modification of GCDH by glutarylation suppresses its activity, while SIRT5 restores its function via deglutarylation [30]. From a broader perspective, as noted by the authors of the review [68], glutarylation belongs to a family of acidic lysine modifications (alongside malonylation and succinylation) which, despite their structural similarity, have different molecular targets and act in various cellular pathways. SIRT5 removes all three modifications, thus playing a central role in the control of metabolic protein function, mitochondrial health, and cellular energy balance.

### Deacetylation

It is well-established that the sirtuin family can modulate the acetylation profile of histone and non-histone proteins [71]. However, the influence of individual members of the family is not uniform. Indeed, SIRT5 activity in this regard is low [72]. Nevertheless, as an NAD⁺-dependent deacetylase, SIRT5 influences the survival of cancer cells by modifying promyelocytic leukemia protein (PML), a suppressor protein whose acetylation regulates cell survival under oxidative stress conditions [73]. In the presence of H₂O₂, SIRT5 (together with SIRT1) promotes the deacetylation of PML, leading to the accumulation of its nuclear form and increased cancer cell death. Furthermore, SIRT5 also acts as an activator of urate oxidase (UOX) through its deacetylation, increasing the enzymatic activity of UOX in liver mitochondria, suggesting its involvement in purine metabolism [18]. Interestingly, SIRT5 activities may overlap. SIRT5 removes succinyl groups in LPS-activated macrophages and regulates PKM2 function, reducing its pyruvate kinase activity and inhibiting the formation of IL-1β. At the same time, the absence of SIRT5 promotes the development of colitis in mice [20]. Additionally, SIRT5 also controls pyruvate metabolism in mitochondria by deacetylating STAT3, limiting its function in converting pyruvate to acetyl-CoA and ATP production in lung cancer cells [21].

## The role of SIRT5 in regulating heart function

The growth and development of fetuses are determined not only by morphological and histological changes in the heart, but also by profound metabolic shifts [74]. One of the most critical changes is the metabolic transition from glycolysis to fatty acid oxidation as the primary energy source [75]. This involves the activation of β-oxidation by releasing inhibitory mechanisms [75]. In rabbits (in a model of heart metabolic development), age-dependent reduction of SIRT5 expression has been observed, along with increased succinylation of key mitochondrial proteins such as pyruvate dehydrogenase complex (PDH) and long-chain specific acyl-CoA dehydrogenase (LCAD) [76].

SIRT5 and lysine succinylation are essential for maintaining cardiac metabolic balance. Their disruption leads to impaired cardiac function, as evidenced by reduced ejection fraction and fractional shortening [77]. This is accompanied by mitochondrial dysfunction, including decreased ECHA activity, accumulation of long-chain acyl-CoA, and impaired fatty acid oxidation, resulting in reduced ATP production [77, 78]. Under oxidative stress, SIRT5 is significantly downregulated, which decreases cell viability and increases apoptosis through enhanced caspase 3/7 activity [79].

Beyond metabolic regulation, SIRT5 directly interacts with Bcl-Xl, modulating its anti-apoptotic role; however, this effect is absent in Bcl-Xl-deficient cells [79]. Moreover, SIRT5 maintains redox homeostasis by activating NADPH-generating enzymes, including IDH2 and G6PD, through desuccinylation and deglutarylation, respectively [80]. Adaptive stress conditions in cardiomyocytes, such as intermittent hypoxia, induce SIRT5 expression, enhancing energy metabolism enzyme levels [81]. Additionally, SIRT5 deficiency impairs mitochondrial ATP production and activates the AMPK pathway [82]. While broad acylome remodeling (e.g., hypermalonylation) may have a limited impact under normal conditions, under severe energy stress (e.g., SLC25A3 deletion), SIRT5 function is compromised by acetylation, exacerbating mitochondrial dysfunction in the heart [83, 84].

These findings highlight the multifaceted role of SIRT5 in cardiac development and lifelong heart function, positioning it as a key regulator of mitochondrial homeostasis, energy metabolism, and cellular resilience.

## Sirtuin 5 and cardiovascular disease

Hypertension, myocardial infarction, atrial fibrillation, diabetic cardiomyopathy, stroke, thrombotic disorders, heart failure, vascular diseases, cardiac hypertrophy, and ischemia-reperfusion injury frequently intersect in clinical practice. These conditions rarely occur in isolation; instead, they form a dangerous synergy that worsens outcomes. Uncontrolled hypertension strains the heart, leading to left ventricular hypertrophy. This structural change sets the stage for atrial fibrillation, which dramatically elevates stroke risk. Similarly, diabetes does not just affect blood sugar, but it reshapes cardiac tissue, while simultaneously promoting thrombotic events that can trigger myocardial infarction or embolic strokes. At the molecular level, proteins like SIRT5 emerge as potential mediators of these pathological connections. By regulating cellular metabolism, oxidative responses, and programmed cell death, such molecules may influence how one condition predisposes one to another. This interconnectedness explains why treating cardiovascular diseases in isolation often proves inadequate and why therapeutic strategies targeting shared pathways hold particular promise.

### SIRT5 and vessels

Blood vessel damage in different diseases has surprisingly similar mechanisms, including metabolic disorders and PTMs of proteins [85]. The rarity of capillaries and the accompanying hypertension correlate with mitochondrial dysfunction in endothelial cells and reduced SIRT5 expression. This process is based on excessive ROS production and impaired angiogenesis [86]. Under microgravity conditions, increased protein succinylation in endothelial cells induces apoptosis through modification of ERO1A (a key protein involved in endoplasmic reticulum stress) [87]. In turn, in aortic aneurysms and dissections, a general increase in lysine succinylation is observed, particularly in proteins associated with energy metabolism, such as OXCT1 [88, 89]. These findings suggest that disturbances in protein succinylation homeostasis, regardless of their cause, may represent a common pathway in various vascular pathologies, contributing to endothelial dysfunction, weakened vascular wall integrity, and impaired microcirculation repair.

### SIRT5 and ischemia-reperfusion injury

SIRT5 is emerging as a key guardian of energetic homeostasis in ischemia-reperfusion (I/R) injury, a process in which the restoration of blood flow provokes a surge of mitochondrial reactive oxygen species (ROS) and tissue damage [90]. A central mediator of this response is succinate, which accumulates during ischemia and is rapidly oxidized upon reperfusion, driving ROS production via reverse electron transport at complex I [90, 91]. As demonstrated by Liu et al., prolonged NAD⁺ supplementation (14 days) enhances SIRT5–SDH coupling and limits ROS generation, indicating that SIRT5-mediated protection arises from pre-reperfusion modulation of succinate metabolism rather than acute enzymatic regulation [91]. Proteomic studies show that loss of SIRT5 leads to increased succinylation of mitochondrial proteins, including nucleotide transporters and β-oxidation enzymes, and correlates with larger infarct size in Sirt5⁻/⁻ hearts [92]. Interestingly, exogenous NAD⁺ administration does not alter SIRT5 or SDH expression levels but enhances their interaction, promotes SDH desuccinylation, and reduces metabolic toxicity and oxidative stress [91]. However, SIRT5’s role is not unidirectional. In failing human myocardium, succinylation of myofibrillar proteins is globally reduced, potentially reflecting limited succinyl-CoA pools and more complex post-ischemic regulation [93]. Additionally, in murine models, hepatic SIRT5 chronic overexpression confers cardioprotection after myocardial infarction by increasing FGF21 levels and activating mitochondrial energy pathways [94]. Meanwhile, in adipose-derived mesenchymal stem cells, SIRT5 deficiency paradoxically enhances regenerative capacity under ischemic conditions by increasing succinylation and shifting metabolism toward glycolysis [95]. Further studies have shown that SIRT5 coordinates mitochondrial interactions by stabilizing the ANT2–VDAC1 complex. Local SIRT5 upregulation in the heart prevents ANT2 lactylation, thereby maintaining mitochondrial integrity and improving cardiac function after I/R [96]. Similarly, SIRT5 mediates the protective effects of quercetin by engaging the DNA–PKcs–SIRT5 axis to regulate mitophagy and the unfolded protein response (UPRmt) [97]. Comparable cardiac protection results were observed in both mice with acute cardiac-specific SIRT5 overexpression and mice treated with long-term quercetin, indicating that sustained activation of the DNA-PKcs-SIRT5 axis provides similar mitochondrial and anti-apoptotic benefits during ischemic-reperfusion injury [97]. Notably, SIRT5 also indirectly influences the stability of MG53, a membrane repair protein, by modulating its succinylation and ubiquitination status during I/R [98]. Does SIRT5 have an unconditional protective effect? The answer depends on the context, protecting mitochondrial function in the heart, but its suppression may enhance regenerative or therapeutic capabilities. This biochemical paradox makes SIRT5 an unobvious therapeutic target.

### SIRT5 and atrial fibrillation

Atrial fibrillation (AF), the most common arrhythmia in adults, is not only an electrical disorder but also a condition characterized by profound metabolic remodeling in atrial tissue cells [99]. Proteomic studies have shown a significant reduction in the levels of many key enzymes involved in carbohydrate metabolism, lipid processing, and oxidative phosphorylation in the left atrial tissue of patients with valvular AF [100]. The molecular link between heart failure (HF) and AF involves mitochondrial dysfunction in the atria, leading to impaired energy metabolism and oxidative stress that promote the development of both conditions [101]. In this context, the role of PTMs, especially lysine succinylation regulated by SIRT5, becomes particularly intriguing. In the atrial tissue of patients with persistent AF, significant changes in the succinylation status of proteins associated with energy metabolism, cell structure, and extracellular matrix organization have been observed [102]. These modifications do not appear to be mere byproducts of AF; on the contrary, they may actively initiate or perpetuate atrial remodeling conducive to arrhythmia. Compelling evidence comes from experimental models of succinate overload, a metabolic precursor that intensifies succinylation. Excess succinate promotes atrial dilation, connexin lateralization, ion channel dysfunction, and oxidative stress, thereby increasing AF susceptibility in both in vivo and in vitro systems [103]. Mechanistically, succinate activates its receptor SUCNR1 while suppressing phosphorylation of AMP-activated protein kinase (AMPK), which compromises mitochondrial function, mirroring the metabolic signatures observed in human AF [103]. In summary, despite many discoveries in the field of epigenetic regulation of the new axis in AF pathophysiology, it remains a mystery whether it is merely a passive observer of metabolic and energy chaos in AF or a decisive moderator.

### SIRT5 and thrombosis

Emerging evidence positions SIRT5 as a pivotal regulator of thrombosis and microembolization, with its effects spanning metabolic reprogramming, endothelial function, and fibrinolysis. In cardioembolic (CE) stroke, elevated succinate, a metabolite linked to SIRT5’s desuccinylation activity, is strongly associated with atrial dysfunction, left atrial enlargement, and stroke recurrence, suggesting a metabolic bridge between atrial pathology and thromboembolism [104]. This aligns with coronary microembolization (CME) findings, where reduced succinyl-CoA levels impair TRMT10C succinylation, diverting this mitochondrial protein to the nucleus. Nuclear TRMT10C triggers m1A-mediated decay of TAFAZZIN and NLRX1, exacerbating inflammation, ROS production, and mitophagy suppression, key drivers of microvascular dysfunction [105]. Paradoxically, the role of SIRT5 in arterial thrombosis reveals a contrasting mechanism: overexpression of transgenic SIRT5 accelerates clot formation after endothelial damage, while SIRT5 knockout attenuates thrombosis in patients with acute coronary syndrome by inhibiting plasminogen activator inhibitor-1 (PAI-1) via AMPK/ERK signaling. Notably, this effect is independent of platelet function or coagulation cascade activation, highlighting SIRT5’s endothelial-specific influence on fibrinolysis [106]. These studies collectively paint SIRT5 as a metabolic orchestrator with context-dependent outcomes: while its dysregulation promotes CE stroke and CME through mitochondrial and metabolic disruptions [104, 105], its overexpression exacerbates arterial thrombosis via PAI-1-mediated fibrinolysis inhibition [106].

### SIRT5 and stroke

Although sirtuins are mainly known as guardians of mitochondrial homeostasis, SIRT5, which has been marginalized in research on the central nervous system, reveals its ambiguous yet crucial impact on the course of ischaemic stroke. It has been shown in experimental models of stroke that SIRT5 deficiency leads to increased brain damage, increased infarct volume, and worsened neurological deficits, which are associated with uncontrolled protein succinylation [107]. At the same time, in subarachnoid hemorrhage, SIRT5 protects mitochondria from oxidative stress: its desuccinylase activity restores the function of key enzymes such as citrate synthase and ATP synthase, reducing ROS production and neuronal death [108]. However, it does not always act as a protector. In a study about microglia, SIRT5 may paradoxically exacerbate the neuroinflammatory cascade. Degradation of SIRT5 by the Tat-SIRT5-CTM peptide reduced microglial activity, limited the infarct area, and improved cognitive function in mice after stroke [109]. In turn, the selective SIRT5 inhibitor (MC3482) had a similar effect, increasing ANXA1 succinylation, inhibiting inflammation, and improving neurological outcomes [110, 111]. Mechanistically, SIRT5 desuccinylates ANXA1, limiting its recruitment to membranes and secretion, which promotes microglial activation and neuronal damage [111]. Furthermore, its function is not limited to microglial cells. Cerebral ischemia-reperfusion injury compromises the integrity of the blood-brain barrier, leading to the degradation of essential tight junction proteins, such as occludin; studies have shown a notable reduction in occludin levels in SIRT5 knockout models following such injuries [10, 112]. In brain endothelial cells, SIRT5 silencing improves barrier integrity through activation of the PI3K/Akt pathway and increased levels of cadherin-5 [112]. Additionally, SIRT5 regulates ferroptosis, iron-dependent cell death, through the Nrf2/HO-1 signaling axis, and its silencing in stroke models reduces oxidative stress, inflammation, and lesion volume [113]. Surprisingly, although SIRT5 appears to protect mitochondria and brain energy, its presence in microglia and endothelium may contribute to damage in the acute phases of stroke. The role of SIRT5 in modulating metabolic, inflammatory, and cell death pathways makes it a fascinating target for future therapeutic interventions in stroke.

### SIRT5 and cardiac hypertrophy

Cardiac hypertrophy is a condition in which the heart increases its muscle mass in response to internal or external stress, which has serious metabolic consequences [114]. Growing evidence suggests that SIRT5 may play a modulatory role in the progression of pathological hypertrophy, potentially influencing the metabolic and mitochondrial stress pathways implicated in the development of heart failure. Although its role in whole-body metabolic regulation remains incompletely understood [11], SIRT5 is abundantly expressed in the heart, suggesting a specialized function in cellular stress responses. Local overexpression of SIRT5 in cardiomyocytes protects against hypertrophy and mitochondrial dysfunction, while global loss of SIRT5 throughout the body leads to cumulative metabolic disorders, excessive protein succinylation, exacerbated hypertrophy, and accelerated heart failure (Table 3). Mice with global SIRT5KO display increased mortality following TAC, accompanied by exacerbated cardiac hypertrophy and impaired systolic function. These phenotypes are closely associated with profound deficits in oxidative metabolism, including reduced fatty acid and glucose oxidation and a lower mitochondrial NAD⁺/NADH ratio [11]. Interestingly, when SIRT5 deletion is restricted to the heart, survival rates remain unaffected compared to controls, despite marked accumulation of succinylated mitochondrial proteins, indicating that SIRT5’s cardioprotective effects may be systemic or developmental [118]. Conversely, SIRT5 overexpression confers protection against TAC-induced pathology by preventing left ventricular dilation and reduced ejection fraction, while preserving oxidative metabolism and suppressing inflammation and fibrosis [119]. These data perfectly correspond to the increased chamber stiffness and impaired relaxation accompanying SIRT5KO [11]. In the context of PTMs, SIRT5 also acts as a demalonylase. Notably, lysine malonylation, previously considered marginal, was found to be significantly reduced in hypertrophic hearts, particularly affecting key metabolic enzymes such as isocitrate dehydrogenase 2 (IDH2), with possible functional consequences despite unchanged protein levels [120]. SIRT5 additionally counteracts the metabolic reprogramming of cardiac fibroblasts toward glycolysis. Metabolic reprogramming, in cardiac cells, accompanies various physiological states, such as cardiac remodeling in newborns due to changes in blood oxygenation, and in the adult heart due to disease [121, 122]. This effect is mediated through the regulation of succinylation of phosphoenolpyruvate carboxykinase 2 (PCK2), a rate-limiting glycolytic enzyme. SIRT5 deficiency leads to increased fibroblast proliferation and collagen synthesis, thereby worsening cardiac fibrosis [123]. Furthermore, metabolic remodeling coupled with mitochondrial dysfunction leads to energy starvation in the failing heart. SIRT5 appears to interact functionally with other mitochondrial remodeling regulators such as HINT2. Overexpression of HINT2 reduces cardiomyocyte hypertrophy and improves mitochondrial function, whereas its loss exacerbates pathological phenotypes [116]. Finally, recent research demonstrates that the transcriptional corepressor RIP140 inhibits SIRT5 expression, which in turn disrupts mitochondrial membrane potential, reduces ATP levels, and exacerbates hypertrophy [117]. Collectively, these findings weave a compelling narrative: SIRT5 emerges as not only a modulator of PTMs but a central regulator of metabolic integrity and survival under stress.

**Table 3:** Comparison of systemic and local effects of SIRT5 modulation

Model	Type	Main findings	Hypertrophic effect	Impact on HF progression	Ref
Global SIRT5-KO	Whole-body knockout	Loss of SIRT5 exacerbates pressure overload–induced hypertrophy and accelerates the transition to heart failure	Severe hypertrophy, no effect of nobiletin	Accelerated HF progression	[115]
Cardiac-specific inducible SIRT5-KO	Cardiomyocyte-specific deletion (adult, inducible)	Regional SIRT5 loss promotes maladaptive remodeling under stress (TAC, MI), associated with higher oxidative stress and mitochondrial dysfunction	Moderate hypertrophy, local dysfunction	Gradual progression of HF	[11]
Global SIRT5 overexpression	Whole-body overexpression	SIRT5-TG mice show resistance to pressure overload–induced hypertrophy and maintain mitochondrial respiration	Complete protection against TAC	Inhibited HF progression	[116]
Regional/cellular SIRT5 overexpression	Neonatal rat cardiomyocytes (in vitro)	SIRT5 overexpression attenuates AngII- or RIP140-induced hypertrophy	Limited hypertrophy after AngII	Provides local protection without systemic effects	[117]

### SIRT5 and myocardial infarction

**Fig. 3:**
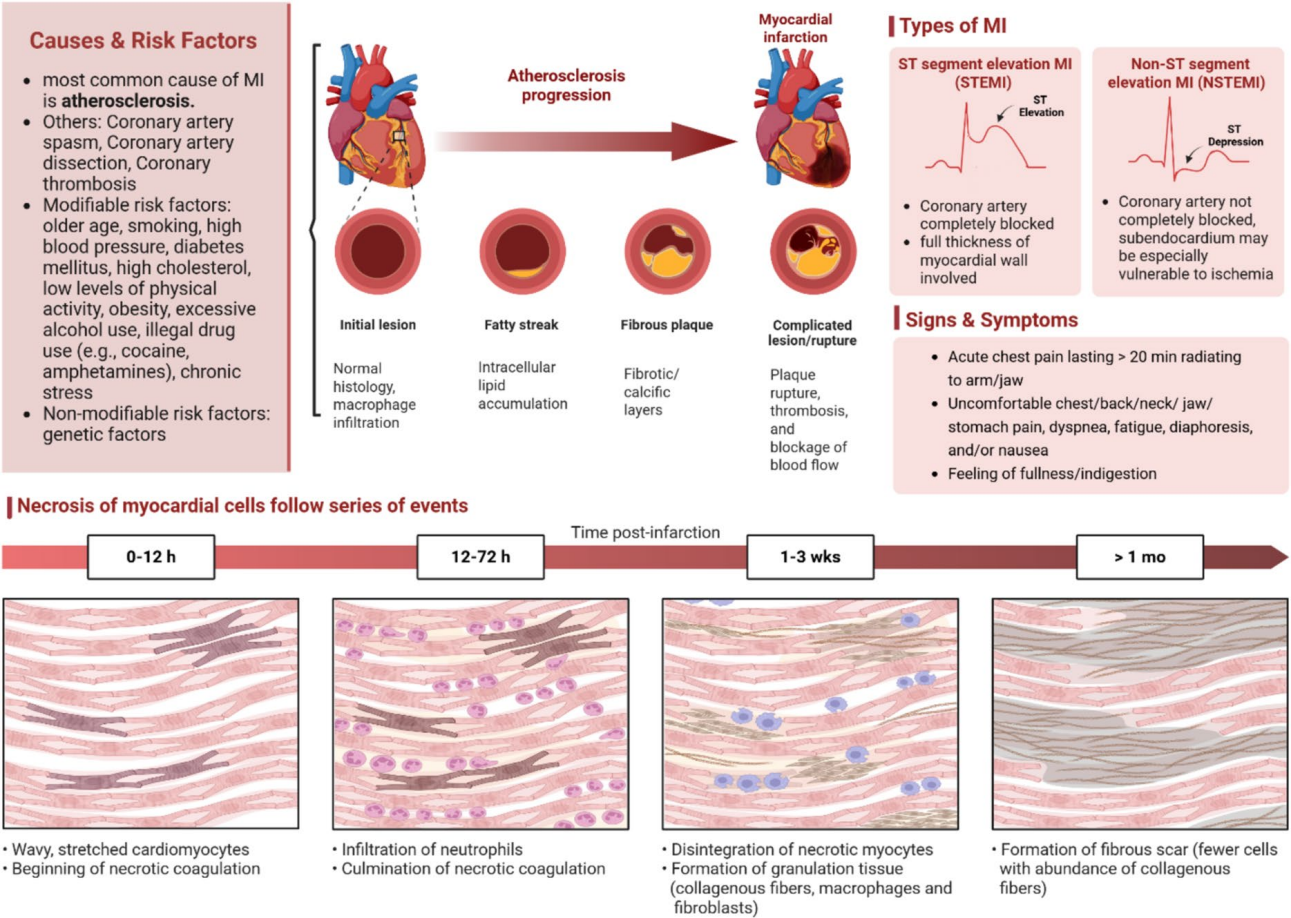
Scheme showing the cause, risk factors, types, and symptoms of myocardial infarction (MI). In the lower part of the scheme, the development of necrosis of myocardial cells is shown. Reference [130]

In light of the central role played by oxidative stress and mitochondrial dysfunction in myocardial damage during infarction (MI), SIRT5 is increasingly viewed as a metabolic regulator and a potential protector of cellular integrity (Fig. 3). Although the genetic basis of coronary artery disease, one of the main factors of MI, remains incomplete, population studies suggest that rare DNA sequence variants in the SIRT5 gene promoter may reduce its transcription and potentially increase the risk of acute coronary syndrome [124]. On a biochemical level, proteomic analyses of serum from MI patients show a significant reduction in protein succinylation and glutarylation, particularly in albumin, which may reflect dysregulated acylation pathways controlled by sirtuins [125]. At the molecular level, SIRT5 exhibits cytoprotective properties. In I/R models, overexpression of SIRT5 immediately prior to ischemia enhances autophagy and reduces cardiomyocyte death through desuccinylation and stabilization of TOM1, a transport protein critical for cell survival [126]. Pharmacologically, catalpol (CAT), a plant-derived compound, improves metabolic indicators and suppresses inflammation in cardiomyocytes. However, these effects are abolished upon SIRT5 knockdown [127]. Interestingly, traditional Chinese compounds such as Zishen Huoxue decoction (ZSHX) also engage mitochondrial defense mechanisms via the SIRT5–β–tubulin axis. ZSHX was shown to modulate mitophagy and the mitochondrial unfolded protein response, suggesting that SIRT5 may serve as a central regulator of cardiomyocyte resistance to hypoxic stress [128]. In a similar manner, activating SIRT5 pharmacologically, still in the ischemic phase, with the agonist MC3215 has been found to limit infarct size and support cardiomyocyte survival. This protective effect appears to involve enhanced mitochondrial fusion through increased Mitofusin 2 levels, as well as the engagement of key metabolic and pro-survival pathways, including AMPK, Akt, and GSK3β [129]. Current data are promising but underscore the need for further research into cell-type specificity and temporal effects during MI progression.

### SIRT5 and diabetic cardiomyopathy

SIRT5, a mitochondrial desuccinylase and demalonylase, is gaining prominence in the context of diabetic cardiomyopathy (DCM). One study showed an age-dependent increase in SIRT5 in patients with type II diabetes. In addition, a causal relationship between SIRT5 expression and diabetes characteristics was demonstrated [131]. SIRT5 expression was positively associated with glucose levels, and reduced expression promoted insulin secretion and pancreatic β-cell proliferation in vitro [131]. Interestingly, in a mouse model of diabetes treated with metformin, it was demonstrated that increased desuccinylation of the alpha subunit of the tripartite enzyme (ECHA) by SIRT5 led to improved glucolipid metabolism [131]. Studies indicate that the expression of sirtuins is significantly disrupted in the diabetic heart. Notably, SIRT5 levels are reduced in the hearts of T1DM rats, but respond positively to resveratrol supplementation, a known activator of SIRT1, highlighting the regulatory plasticity and therapeutic potential of SIRT5 [132]. Furthermore, SIRT5 deficiency exacerbates oxidative stress, DNA damage, and cardiomyocyte senescence in DCM, primarily through impaired demalonylation of GSTP1, a key detoxification protein. Reduced stability of GSTP1 due to excessive lysine malonylation contributes to myocardial injury, whereas SIRT5 overexpression alleviates these effects, an outcome dependent on the transcription factor SPI1, which transcriptionally activates SIRT5 [133].

One of the factors contributing to the deterioration of myocardial function in DCM is the accumulation of fatty acids, which induce lipotoxicity in the heart. Diving deeper into metabolic regulation, SIRT5 is also a critical modulator of FAO in the diabetic heart. In KO-SIRT mice, its absence does not impair fatty acid uptake; it significantly disrupts lipid metabolism by increasing succinylation of carnitine palmitoyltransferase 2 (CPT2), an enzyme responsible for converting fatty acyl-carnitines back to fatty acyl-CoA. A K424R mutation in CPT2, which prevents succinylation, effectively reverses the enzymatic dysfunction caused by SIRT5 loss, thereby reducing lipotoxicity and improving cardiac performance [134].

### SIRT5 and heart failure

Heart failure (HF) is one of the leading causes of morbidity and mortality worldwide. The progressive decline in cardiac systolic and diastolic function is a direct result of drastic disturbances in energy metabolism [135, 136]. Mitochondria, essential for cardiac energy metabolism, become structurally and functionally impaired during HF, with oxidative stress, disrupted biogenesis, and faulty quality control mechanisms driving disease progression [137]. Intriguingly, recent studies have identified SIRT5 as a potential guardian of this delicate system. It has been revealed that pressure overload induced by transverse aortic constriction suppresses SIRT5 expression, leading to increased succinylation of the mitochondrial enzyme IDH2 and disruption of mitochondrial homeostasis [49]. Additionally, under hyperglycemic stress, there was an excess generation of ROS, dysfunction of respiratory complexes I and III, and activation of inflammatory pathways. These effects were mitigated by quercetin, which enhanced SIRT5 expression and restored mitochondrial balance [49]. Parallel findings from a MI model of HF demonstrated significant disturbances in succinyl-CoA metabolism. Its decreased levels impaired oxidative phosphorylation (OXPHOS) and contributed to heart failure progression [138]. Interestingly, supplementation with 5-aminolevulinic acid, a precursor in heme synthesis, partially reversed this defect [138]. In both cases (MI and pressure overload), SIRT5 desuccinylase activity, enhanced by nobiletin, inhibits the expression of hypertrophic genes. In the MI model, its action focuses on limiting post-infarction remodeling, while in TAC, it focuses on modulating the heart’s adaptation to long-term pressure overload, which together leads to a slower transition from acute myocardial damage to chronic HF [115]. Meanwhile, it has been shown that nobiletin, a natural flavonoid, binds directly to SIRT5, enhancing its desuccinylase activity against p300 (a transcription factor), and protects against HF progression [115]. Despite promising preclinical results, SIRT5 remains outside the scope of current HF therapies due to the superficial nature of the knowledge gained about SIRT5 to date. Nevertheless, corresponding results obtained in different models emphasize its role as a stabilizer of mitochondrial function. As has been highlighted, SIRT5 does not act as a central switch but supports metabolic balance under stressful conditions. In the heart with energy disturbances, even such subtle control may represent a significant therapeutic benefit. AI-drug discovery could be used in future research to investigate Sirtuin 5 as a target [139].

## Mechanistic framework for the dual role of SIRT5

The dual role of SIRT5 in cardiovascular and metabolic pathologies can be understood through a unified model that integrates cell type, metabolic state, and disease stage. As a mitochondrial desuccinylase, demalonylase, and deglutarylase, SIRT5 maintains oxidative metabolism and redox balance under conditions of acute stress by maintaining the activity of key mitochondrial enzymes such as SDH and ATP synthase [90, 91, 94]. This allows the accumulation of ROS to be reduced in tissues with high energy demands, such as cardiomyocytes, hepatocytes, or neurons, where SIRT5 supports ATP production. Therefore, in the aforementioned diseases, i.e., ischemia-reperfusion, myocardial infarction, or diabetic cardiomyopathy, it prevents apoptosis signaling [90, 91, 133, 134]. In such situations, its loss causes excessive protein acylation, thereby exacerbating tissue damage, from ATP insufficiency [49, 92].

However, the same metabolism can become maladaptive in contexts requiring metabolic reprogramming or controlled inflammatory activation. In endothelial cells, age-related overexpression of SIRT5 impairs NO bioavailability by inhibiting the eNOS pathway, contributing to vascular stiffness and endothelial dysfunction [48, 86]. In thrombosis, upregulation of SIRT5 accelerates clot formation by activating the AMPK/ERK–PAI-1 axis, thereby reducing fibrinolysis independently of platelet aggregation [106]. Furthermore, SIRT5 deficiency in mesenchymal stem cells increases their glycolytic metabolism and regenerative potential under hypoxic conditions, suggesting that temporary inhibition of SIRT5 may promote tissue repair [95].

In summary, SIRT5 acts as a metabolic stabilizer that has a protective effect in situations requiring high energy but can be potentially harmful when inflammation needs to subside, e.g., in the case of increasing concentrations of inflammatory factors with age, or during tissue repair [140].

The possibility of this context-dependent behavior reconciles the paradoxical role of SIRT5 and points to its potential as a therapeutic target, either through activation to stabilize mitochondrial metabolism or through inhibition to promote adaptive remodeling.

## Outlook and potential future research directions

The future of SIRT5 research in cardiovascular health should be characterized by significant technological and conceptual advancements. Single-cell and spatial multi-omics technologies are poised to delineate SIRT5’s cell–type–specific roles, elucidating how its metabolic and deacetylase functions vary across diverse cardiac cell populations, including cardiomyocytes, fibroblasts, endothelial cells, and immune cells. These methods could unveil intricate transcriptional or metabolic networks that underpin SIRT5’s contribution to myocardial resilience during aging. The implications of SIRT5 in age-related cardiac pathologies are particularly compelling. Given that age-associated declines in mitochondrial efficiency and redox balance drive cardiovascular dysfunction, comprehending SIRT5’s modulation of acylation patterns could inform therapeutic strategies to counteract metabolic inflexibility and fibrosis. However, investigations must extend beyond cardiomyocytes to explore SIRT5’s underexplored influence in cardiac non-myocytes, where it may regulate extracellular matrix remodeling, angiogenesis, and inflammatory signaling. Moreover, emerging evidence suggests potential sex-dependent differences in SIRT5-mediated cardiac protection, highlighting the necessity for experimental designs that balance sex representation and incorporate integrative analyses across hormonal and metabolic axes. The quantification of circulating or myocardial SIRT5 as a biomarker could enhance the early detection of metabolic cardiac stress and predict treatment efficacy. Progress in this domain will be facilitated by collaborative endeavors integrating systems biology, advanced imaging, and translational cardiology. The integration of computational modeling with in vivo and human-derived data will be crucial for defining how SIRT5 orchestrates cardiac homeostasis throughout the lifespan, ultimately enabling precision interventions for age-associated heart disease.

## Conclusion

SIRT5, as a key regulator of mitochondrial metabolism and PTMs, plays a dual role in the pathophysiology of cardiovascular diseases. On the one hand, its desuccinylating and demalonylating activity protects cardiomyocytes from oxidative stress, improves energy efficiency, and inhibits adverse cardiac remodeling. On the other hand, in certain contexts (e.g., in post-stroke microglia or thrombogenic endothelial cells), excessive SIRT5 activity may exacerbate pathological processes.

These conflicting observations indicate that therapeutic modulation of SIRT5 requires a precise, tissue-specific approach. There are promising reports of natural compounds (e.g., quercetin, nobiletin) and pharmacological modulators of SIRT5 that may selectively influence its activity. However, the full therapeutic potential of SIRT5 in cardiology requires a deeper understanding of its metabolic relationships and interactions with other signaling pathways. Ultimately, SIRT5 is not a simple metabolic “switch,” but rather a subtle coordinator of cellular homeostasis, whose role must be considered in the context of a specific disease, its stage of development, and the overall metabolic state of the organism. This makes research on SIRT5 both challenging and an extremely promising direction for developing new strategies for treating heart disease.

## References

[1] Azevedo PS, Minicucci MF, Santos PP, Paiva SAR, Zornoff LAM. Energy metabolism in cardiac remodeling and heart failure. Cardiol Rev. 2013;21:135. 10.1097/CRD.0b013e318274956d. 22990373 10.1097/CRD.0b013e318274956d

[2] Sorescu D, Griendling KK. Reactive oxygen species, mitochondria, and NAD(P)H oxidases in the development and progression of heart failure. Congest Heart Fail. 2002;8:132–40. 10.1111/j.1527-5299.2002.00717.x. 12045381 10.1111/j.1527-5299.2002.00717.x

[3] Lehman JJ, Kelly DP. Gene regulatory mechanisms governing energy metabolism during cardiac hypertrophic growth. Heart Fail Rev. 2002;7:175–85. 10.1023/A:1015332726303. 11988641 10.1023/a:1015332726303

[4] Cheng X, Wang K, Zhao Y, Wang K. Research progress on post-translational modification of proteins and cardiovascular diseases. Cell Death Discov. 2023;9:1–12. 10.1038/s41420-023-01560-5. 37507372 10.1038/s41420-023-01560-5 PMC10382489

[5] Morita Y, Tohyama S. Metabolic regulation of cardiac differentiation and maturation in pluripotent stem cells: a lesson from heart development. JMA J. 2020;3:193–200. 10.31662/jmaj.2020-0036. 33150253 10.31662/jmaj.2020-0036 PMC7590396

[6] Lopaschuk GD, Karwi QG, Tian R, Wende AR, Abel ED. Cardiac energy metabolism in heart failure. Circ Res. 2021;128:1487–513. 10.1161/CIRCRESAHA.121.318241. 33983836 10.1161/CIRCRESAHA.121.318241 PMC8136750

[7] Wu Q-J, Zhang T-N, Chen H-H, Yu X-F, Lv J-L, Liu Y-Y, et al. The sirtuin family in health and disease. Signal Transduct Target Ther. 2022;7:1–74. 10.1038/s41392-022-01257-8. 36581622 10.1038/s41392-022-01257-8 PMC9797940

[8] Grzeczka A, Skowronska A, Sepe S, Skowronski MT, Kordowitzki P. Sirtuins and their role in ovarian aging-related fibrosis predisposing to ovarian cancer. NPJ Aging. 2025;11:65. 10.1038/s41514-025-00256-7. 40664665 10.1038/s41514-025-00256-7 PMC12264197

[9] Wang Y, Chen H, Zha X. Overview of SIRT5 as a potential therapeutic target: structure, function and inhibitors. Eur J Med Chem. 2022;236:114363. 10.1016/j.ejmech.2022.114363. 35436671 10.1016/j.ejmech.2022.114363

[10] Satoh K, Shimokawa H. Sirtuin 5 promotes ischemia/reperfusion-induced blood-brain barrier damage after stroke. Int J Cardiol. 2019;284:77–8. 10.1016/j.ijcard.2018.11.019. 30448019 10.1016/j.ijcard.2018.11.019

[11] Hershberger KA, Abraham DM, Martin AS, Mao L, Liu J, Gu H, et al. Sirtuin 5 is required for mouse survival in response to cardiac pressure overload. J Biol Chem. 2017;292:19767–81. 28972174 10.1074/jbc.M117.809897 PMC5712617

[12] Chen L, Wang H, Gao F, Zhang J, Zhang Y, Ma R, et al. Functional genetic variants in the *SIRT5* gene promoter in acute myocardial infarction. Gene. 2018;675:233–9. 10.1016/j.gene.2018.07.010. 29981421 10.1016/j.gene.2018.07.010

[13] Yang X, Liu B, Zhu W, Luo J. SIRT5, functions in cellular metabolism with a multiple enzymatic activities. Sci China Life Sci. 2015;58:912–4. 10.1007/s11427-015-4902-8. 26208827 10.1007/s11427-015-4902-8

[14] Nakagawa T, Lomb DJ, Haigis MC, Guarente L. SIRT5 deacetylates carbamoyl phosphate synthetase 1 and regulates the urea cycle. Cell. 2009;137:560–70. 10.1016/j.cell.2009.02.026. 19410549 10.1016/j.cell.2009.02.026 PMC2698666

[15] Tan M, Peng C, Anderson KA, Chhoy P, Xie Z, Dai L, et al. Lysine glutarylation is a protein posttranslational modification regulated by SIRT5. Cell Metab. 2014;19:605–17. 10.1016/j.cmet.2014.03.014. 24703693 10.1016/j.cmet.2014.03.014 PMC4108075

[16] Polletta L, Vernucci E, Carnevale I, Arcangeli T, Rotili D, Palmerio S, et al. SIRT5 regulation of ammonia-induced autophagy and mitophagy. Autophagy. 2015;11:253–70. 10.1080/15548627.2015.1009778. 25700560 10.1080/15548627.2015.1009778 PMC4502726

[17] Yang H, Gao S, Lu G, He J, Dong J, Zhang X, et al. SIRT5-mediated GLS and GDH desuccinylation attenuates the autophagy of bovine mammary epithelial cells induced by ammonia. Cell Signal. 2025;127:111570. 10.1016/j.cellsig.2024.111570. 39694127 10.1016/j.cellsig.2024.111570

[18] Nakamura Y, Ogura M, Ogura K, Tanaka D, Inagaki N. SIRT5 deacetylates and activates urate oxidase in liver mitochondria of mice. FEBS Lett. 2012;586:4076–81. 10.1016/j.febslet.2012.10.009. 23085393 10.1016/j.febslet.2012.10.009

[19] Nishida Y, Rardin MJ, Carrico C, He W, Sahu AK, Gut P, et al. SIRT5 regulates both cytosolic and mitochondrial protein malonylation with glycolysis as a major target. Mol Cell. 2015;59:321–32. 10.1016/j.molcel.2015.05.022. 26073543 10.1016/j.molcel.2015.05.022 PMC4571487

[20] Wang F, Wang K, Xu W, Zhao S, Ye D, Wang Y, et al. SIRT5 desuccinylates and activates pyruvate kinase M2 to block macrophage IL-1β production and to prevent DSS-induced colitis in mice. Cell Rep. 2017;19:2331–44. 10.1016/j.celrep.2017.05.065. 28614718 10.1016/j.celrep.2017.05.065

[21] Xu YS, Liang JJ, Wang Y, Zhao XJ, Xu L, Xu Y, et al. STAT3 undergoes acetylation-dependent mitochondrial translocation to regulate pyruvate metabolism. Sci Rep. 2016;6:39517. 10.1038/srep39517. 28004755 10.1038/srep39517 PMC5177931

[22] Park J, Chen Y, Tishkoff DX, Peng C, Tan M, Dai L, et al. SIRT5-mediated lysine desuccinylation impacts diverse metabolic pathways. Mol Cell. 2013;50:919–30. 10.1016/j.molcel.2013.06.001. 23806337 10.1016/j.molcel.2013.06.001 PMC3769971

[23] Zong Y, Li H, Liao P, Chen L, Pan Y, Zheng Y, et al. Mitochondrial dysfunction: mechanisms and advances in therapy. Signal Transduct Target Ther. 2024;9:124. 10.1038/s41392-024-01839-8. 38744846 10.1038/s41392-024-01839-8 PMC11094169

[24] Teng P, Cui K, Yao S, Fei B, Ling F, Li C, et al. SIRT5-mediated ME2 desuccinylation promotes cancer growth by enhancing mitochondrial respiration. Cell Death Differ. 2024;31:65–77. 38007551 10.1038/s41418-023-01240-y PMC10781994

[25] Zhang Y, Bharathi SS, Rardin MJ, Lu J, Maringer KV, Sims-Lucas S, et al. Lysine desuccinylase SIRT5 binds to cardiolipin and regulates the electron transport chain. J Biol Chem. 2017;292:10239–49. 10.1074/jbc.M117.785022. 28458255 10.1074/jbc.M117.785022 PMC5473227

[26] Rardin MJ, He W, Nishida Y, Newman JC, Carrico C, Danielson SR, et al. SIRT5 regulates the mitochondrial lysine succinylome and metabolic networks. Cell Metab. 2013;18:920–33. 10.1016/j.cmet.2013.11.013. 24315375 10.1016/j.cmet.2013.11.013 PMC4105152

[27] Lin Z-F, Xu H-B, Wang J-Y, Lin Q, Ruan Z, Liu F-B, et al. SIRT5 desuccinylates and activates SOD1 to eliminate ROS. Biochem Biophys Res Commun. 2013;441:191–5. 10.1016/j.bbrc.2013.10.033. 24140062 10.1016/j.bbrc.2013.10.033

[28] He X, Li T, Qin K, Luo S, Li Z, Ji Q, et al. Demalonylation of DDX3 by sirtuin 5 promotes antiviral innate immune responses. Theranostics. 2021;11:7235–46. 10.7150/thno.52934. 34158847 10.7150/thno.52934 PMC8210596

[29] Liu X, Rong F, Tang J, Zhu C, Chen X, Jia S, et al. Repression of P53 function by SIRT5-mediated desuccinylation at lysine 120 in response to DNA damage. Cell Death Differ. 2022;29:722–36. 10.1038/s41418-021-00886-w. 34642466 10.1038/s41418-021-00886-w PMC8989948

[30] Bhatt DP, Mills CA, Anderson KA, Henriques BJ, Lucas TG, Francisco S, et al. Deglutarylation of glutaryl-CoA dehydrogenase by deacylating enzyme SIRT5 promotes lysine oxidation in mice. J Biol Chem. 2022;298:101723. 35157847 10.1016/j.jbc.2022.101723 PMC8969154

[31] Donlon TA, Morris BJ, Chen R, Masaki KH, Allsopp RC, Willcox DC, et al. Analysis of polymorphisms in 59 potential candidate genes for association with human longevity. J Gerontol A Biol Sci Med Sci. 2018;73:1459–64. 10.1093/gerona/glx247. 29300832 10.1093/gerona/glx247 PMC6175033

[32] Rotllan N, Camacho M, Tondo M, Diarte-Añazco EMG, Canyelles M, Méndez-Lara KA, et al. Therapeutic potential of emerging NAD+-increasing strategies for cardiovascular diseases. Antioxidants. 2021;10:1939. 10.3390/antiox10121939. 34943043 10.3390/antiox10121939 PMC8750485

[33] Miwa S, Kashyap S, Chini E, Zglinicki T. Mitochondrial dysfunction in cell senescence and aging. J Clin Invest. 2022. 10.1172/JCI158447. 35775483 10.1172/JCI158447 PMC9246372

[34] Rodríguez-Díaz A, Marín-Conde E, Gómez-Tatay L. Personalist bioethics as a guide to assessing emerging anti-aging therapies. Linacre Q 2025; 00243639251361196. 10.1177/00243639251361196. 10.1177/00243639251361196 PMC12354403 40821382

[35] Campisi J, Kapahi P, Lithgow GJ, Melov S, Newman JC, Verdin E. From discoveries in ageing research to therapeutics for healthy ageing. Nature. 2019;571:183–92. 31292558 10.1038/s41586-019-1365-2 PMC7205183

[36] Picca A, Mankowski RT, Burman JL, Donisi L, Kim J-S, Marzetti E, et al. Mitochondrial quality control mechanisms as molecular targets in cardiac ageing. Nat Rev Cardiol. 2018;15:543–54. 10.1038/s41569-018-0059-z. 30042431 10.1038/s41569-018-0059-z PMC6283278

[37] Haschler TN, Horsley H, Balys M, Anderson G, Taanman J-W, Unwin RJ, et al. Sirtuin 5 depletion impairs mitochondrial function in human proximal tubular epithelial cells. Sci Rep. 2021;11:15510. 10.1038/s41598-021-94185-6. 34330933 10.1038/s41598-021-94185-6 PMC8324880

[38] Walker MA, Tian R. NAD(H) in mitochondrial energy transduction: implications for health and disease. Curr Opin Physiol. 2018;3:101–9. 10.1016/j.cophys.2018.03.011. 32258851 10.1016/j.cophys.2018.03.011 PMC7112453

[39] Ali HR, Michel CR, Lin YH, McKinsey TA, Jeong MY, Ambardekar AV, et al. Defining decreased protein succinylation of failing human cardiac myofibrils in ischemic cardiomyopathy. J Mol Cell Cardiol. 2020;138:304–17. 10.1016/j.yjmcc.2019.11.159. 31836543 10.1016/j.yjmcc.2019.11.159 PMC7058372

[40] Scieszka D, Bolt AM, McCormick MA, Brigman JL, Campen MJ. Aging, longevity, and the role of environmental stressors: a focus on wildfire smoke and air quality. Front Toxicol. 2023. 10.3389/ftox.2023.1267667. 37900096 10.3389/ftox.2023.1267667 PMC10600394

[41] Xin Y, Zhang X, Li J, Gao H, Li J, Li J, et al. New insights into the role of mitochondria quality control in ischemic heart disease. Front Cardiovasc Med. 2021. 10.3389/fcvm.2021.774619. 34901234 10.3389/fcvm.2021.774619 PMC8661033

[42] Luo J, Mills K, le Cessie S, Noordam R, van Heemst D. Ageing, age-related diseases and oxidative stress: what to do next? Ageing Res Rev. 2020;57:100982. 10.1016/j.arr.2019.100982. 31733333 10.1016/j.arr.2019.100982

[43] Hall DJ, Freeman LM, Rush JE, Cunningham SM. Comparison of serum fatty acid concentrations in cats with hypertrophic cardiomyopathy and healthy controls. J Feline Med Surg. 2014;16:631–6. 10.1177/1098612X13516478. 24366844 10.1177/1098612X13516478 PMC11164160

[44] Haigis MC, Sinclair DA. Mammalian sirtuins: biological insights and disease relevance. Annu Rev Pathol Mech Dis. 2010;5:253–95. 10.1146/annurev.pathol.4.110807.092250. 10.1146/annurev.pathol.4.110807.092250 PMC2866163 20078221

[45] Gut P, Matilainen S, Meyer JG, Pällijeff P, Richard J, Carroll CJ, et al. *SUCLA2* mutations cause global protein succinylation contributing to the pathomechanism of a hereditary mitochondrial disease. Nat Commun. 2020;11:5927. 10.1038/s41467-020-19743-4. 33230181 10.1038/s41467-020-19743-4 PMC7684291

[46] Kalous KS, Wynia-Smith SL, Smith BC. Sirtuin oxidative post-translational modifications. Front Physiol. 2021. 10.3389/fphys.2021.763417. 34899389 10.3389/fphys.2021.763417 PMC8652059

[47] Hou X, Zhu L, Xu H, Shi J, Ji S. Dysregulation of protein succinylation and disease development. Front Mol Biosci. 2024. 10.3389/fmolb.2024.1407505. 38882606 10.3389/fmolb.2024.1407505 PMC11176430

[48] Yang K, Akhmedov A, Kraler S, Schmiady M, Puspitasari Y, Camici G, et al. Sirt5 as a mediator of endothelial and vascular dysfunction: potential therapeutic target in aging-related vascular diseases. Eur Heart J. 2024;45:ehae666.3899. 10.1093/eurheartj/ehae666.3899.

[49] Chang X, Zhang T, Wang J, Liu Y, Yan P, Meng Q, et al. SIRT5-related desuccinylation modification contributes to quercetin-induced protection against heart failure and high-glucose-prompted cardiomyocytes injured through regulation of mitochondrial quality surveillance. Oxid Med Cell Longev. 2021;2021:5876841. 10.1155/2021/5876841. 34603599 10.1155/2021/5876841 PMC8486530

[50] Zeng Z, Xu P, He Y, Yi Y, Liu Z, Cai J, et al. Acetylation of Atp5f1c mediates cardiomyocyte senescence via metabolic dysfunction in radiation-induced heart damage. Oxid Med Cell Longev. 2022;2022:4155565. 10.1155/2022/4155565. 36160705 10.1155/2022/4155565 PMC9499811

[51] Chen P-T, Yeong KY. New sirtuin modulators: their uncovering, pharmacophore, and implications in drug discovery. Med Chem Res. 2024;33:1064–78. 10.1007/s00044-024-03249-5.

[52] Giblin W, Bringman-Rodenbarger L, Guo AH, Kumar S, Monovich AC, Mostafa AM, et al. The deacylase SIRT5 supports melanoma viability by influencing chromatin dynamics. J Clin Invest. 2021. 10.1172/JCI138926. 33945506 10.1172/JCI138926 PMC8203465

[53] Bowman CE, Wolfgang MJ. Role of the malonyl-CoA synthetase ACSF3 in mitochondrial metabolism. Adv Biol Regul. 2019;71:34–40. 30201289 10.1016/j.jbior.2018.09.002 PMC6347522

[54] Weinert BT, Schölz C, Wagner SA, Iesmantavicius V, Su D, Daniel JA, et al. Lysine succinylation is a frequently occurring modification in prokaryotes and eukaryotes and extensively overlaps with acetylation. Cell Rep. 2013;4:842–51. 23954790 10.1016/j.celrep.2013.07.024

[55] Fischer F, Gertz M, Suenkel B, Lakshminarasimhan M, Schutkowski M, Steegborn C. Sirt5 deacylation activities show differential sensitivities to nicotinamide inhibition. PLoS One. 2012;7:e45098. 10.1371/journal.pone.0045098. 23028781 10.1371/journal.pone.0045098 PMC3446968

[56] Hang T, Chen W, Wu M, Zhan L, Wang C, Jia N, et al. Structural insights into the molecular mechanism underlying Sirt5-catalyzed desuccinylation of histone peptides. Biochem J. 2019;476:211–23. 30523058 10.1042/BCJ20180745

[57] Ruan J, Zheng J, Zhang X, Chen Z, Sun Y, Jia X. SIRT5 suppresses the trophoblast cell proliferation, invasion, and migration to promote preeclampsia via desuccinylating HOXB3. J Assist Reprod Genet. 2024;41:2759–70. 10.1007/s10815-024-03223-5. 39145876 10.1007/s10815-024-03223-5 PMC11535100

[58] Zhao X, Yang X, Du C, Hao H, Liu S, Liu G, et al. Up-regulated succinylation modifications induce a senescence phenotype in microglia by altering mitochondrial energy metabolism. J Neuroinflammation. 2024;21:296. 10.1186/s12974-024-03284-4. 39543710 10.1186/s12974-024-03284-4 PMC11566524

[59] Zhang X, Ling C, Xiong Z, Gong T, Luo S, Liu X, et al. Desuccinylation of TBK1 by SIRT5 regulates inflammatory response of macrophages in sepsis. Cell Rep. 2024. 10.1016/j.celrep.2024.115060. 39673708 10.1016/j.celrep.2024.115060

[60] Gibson GE, Xu H, Chen H-L, Chen W, Denton TT, Zhang S. Alpha-ketoglutarate dehydrogenase complex-dependent succinylation of proteins in neurons and neuronal cell lines. J Neurochem. 2015;134:86–96. 10.1111/jnc.13096. 25772995 10.1111/jnc.13096 PMC4472501

[61] Wang G, Meyer JG, Cai W, Softic S, Li ME, Verdin E, et al. Regulation of UCP1 and mitochondrial metabolism in brown adipose tissue by reversible succinylation. Mol Cell. 2019;74:844-857.e7. 10.1016/j.molcel.2019.03.021. 31000437 10.1016/j.molcel.2019.03.021 PMC6525068

[62] Kurmi K, Hitosugi S, Wiese EK, Boakye-Agyeman F, Gonsalves WI, Lou Z, et al. Carnitine palmitoyltransferase 1A has a lysine succinyltransferase activity. Cell Rep. 2018;22:1365–73. 10.1016/j.celrep.2018.01.030. 29425493 10.1016/j.celrep.2018.01.030 PMC5826573

[63] Siculella L, Giannotti L, Di Chiara Stanca B, Calcagnile M, Rochira A, Stanca E, et al. Evidence for a negative correlation between human reactive enamine-imine intermediate deaminase A (RIDA) activity and cell proliferation rate: role of lysine succinylation of RIDA. Int J Mol Sci. 2021;22:3804. 10.3390/ijms22083804. 33916919 10.3390/ijms22083804 PMC8067581

[64] Zou L, Yang Y, Wang Z, Fu X, He X, Song J, et al. Lysine malonylation and its links to metabolism and diseases. Aging Dis. 2023;14:84. 36818560 10.14336/AD.2022.0711 PMC9937698

[65] Du J, Zhou Y, Su X, Yu JJ, Khan S, Jiang H, et al. Sirt5 is a NAD-dependent protein lysine demalonylase and desuccinylase. Science. 2011;334:806–9. 10.1126/science.1207861. 22076378 10.1126/science.1207861 PMC3217313

[66] Peng C, Lu Z, Xie Z, Cheng Z, Chen Y, Tan M, et al. The first identification of lysine malonylation substrates and its regulatory enzyme. Mol Cell Proteomics. 2011. 10.1074/mcp.M111.012658. 21908771 10.1074/mcp.M111.012658 PMC3237090

[67] Nahálková J. A new view on functions of the lysine demalonylase activity of SIRT5. Life Sci. 2023;320:121572. 10.1016/j.lfs.2023.121572. 36921688 10.1016/j.lfs.2023.121572

[68] Hirschey MD, Zhao Y. Metabolic regulation by lysine malonylation, succinylation, and glutarylation. Molecular & Cellular Proteomics. 2015;14:2308–15. 10.1074/mcp.R114.046664. 25717114 10.1074/mcp.R114.046664 PMC4563717

[69] Zhang R, Bons J, Scheidemantle G, Liu X, Bielska O, Carrico C, et al. Histone malonylation is regulated by SIRT5 and KAT2A. iScience. 2023. 10.1016/j.isci.2023.106193. 36879797 10.1016/j.isci.2023.106193 PMC9985052

[70] Wang H-L, Chen Y, Wang Y-Q, Tao E-W, Tan J, Liu Q-Q, et al. Sirtuin5 protects colorectal cancer from DNA damage by keeping nucleotide availability. Nat Commun. 2022;13:6121. 10.1038/s41467-022-33903-8. 36253417 10.1038/s41467-022-33903-8 PMC9576705

[71] Chen M, Tan J, Jin Z, Jiang T, Wu J, Yu X. Research progress on sirtuins (SIRTs) family modulators. Biomed Pharmacother. 2024;174:116481. 38522239 10.1016/j.biopha.2024.116481

[72] Xie Y, Cai N, Liu X, He L, Ma Y, Yan C, et al. SIRT5: a potential target for discovering bioactive natural products. J Nat Med. 2025;79:441–64. 10.1007/s11418-024-01871-6. 39979670 10.1007/s11418-024-01871-6 PMC12058867

[73] Guan D, Lim JH, Peng L, Liu Y, Lam M, Seto E, et al. Deacetylation of the tumor suppressor protein PML regulates hydrogen peroxide-induced cell death. Cell Death Dis. 2014;5:e1340–e1340. 10.1038/cddis.2014.185. 25032863 10.1038/cddis.2014.185 PMC4123062

[74] Lopaschuk GD, Jaswal JS. Energy metabolic phenotype of the cardiomyocyte during development, differentiation, and postnatal maturation. J Cardiovasc Pharmacol. 2010;56:130. 10.1097/FJC.0b013e3181e74a14. 20505524 10.1097/FJC.0b013e3181e74a14

[75] Piquereau J, Ventura-Clapier R. Maturation of cardiac energy metabolism during perinatal development. Front Physiol. 2018. 10.3389/fphys.2018.00959. 30072919 10.3389/fphys.2018.00959 PMC6060230

[76] Fukushima A, Alrob OA, Zhang L, Wagg CS, Altamimi T, Rawat S, et al. Acetylation and succinylation contribute to maturational alterations in energy metabolism in the newborn heart. Am J Physiol Heart Circ Physiol. 2016;311:H347–63. 10.1152/ajpheart.00900.2015. 27261364 10.1152/ajpheart.00900.2015

[77] Sadhukhan S, Liu X, Ryu D, Nelson OD, Stupinski JA, Li Z, et al. Metabolomics-assisted proteomics identifies succinylation and SIRT5 as important regulators of cardiac function. Proc Natl Acad Sci U S A. 2016;113:4320–5. 10.1073/pnas.1519858113. 27051063 10.1073/pnas.1519858113 PMC4843474

[78] Tian L, Sun Q, Lv J, Liu S, Zhang K, Li Y, et al. Multi-omics analysis of gestational PM2.5 exposure induces abnormal cardiac metabolism and development in offspring. Ecotoxicol Environ Saf. 2025;299:118416. 10.1016/j.ecoenv.2025.118416. 40440979 10.1016/j.ecoenv.2025.118416

[79] Liu B, Che W, Zheng C, Liu W, Wen J, Fu H, et al. SIRT5: a safeguard against oxidative stress-induced apoptosis in cardiomyocytes. Cell Physiol Biochem. 2013;32:1050–9. 10.1159/000354505. 24192575 10.1159/000354505

[80] Zhou L, Wang F, Sun R, Chen X, Zhang M, Xu Q, et al. SIRT5 promotes IDH2 desuccinylation and G6PD deglutarylation to enhance cellular antioxidant defense. EMBO Rep. 2016;17:811–22. 10.15252/embr.201541643. 27113762 10.15252/embr.201541643 PMC5278614

[81] Zhu W-Z, Wu X-F, Zhang Y, Zhou Z-N. Proteomic analysis of mitochondrial proteins in cardiomyocytes from rats subjected to intermittent hypoxia. Eur J Appl Physiol. 2012;112:1037–46. 10.1007/s00421-011-2050-9. 21735218 10.1007/s00421-011-2050-9

[82] Zhang M, Wu J, Sun R, Tao X, Wang X, Kang Q, et al. SIRT5 deficiency suppresses mitochondrial ATP production and promotes AMPK activation in response to energy stress. PLoS One. 2019;14:e0211796. 10.1371/journal.pone.0211796. 30759120 10.1371/journal.pone.0211796 PMC6373945

[83] Fisher-Wellman KH, Draper JA, Davidson MT, Williams AS, Narowski TM, Slentz DH, et al. Respiratory phenomics across multiple models of protein hyperacylation in cardiac mitochondria reveals a marginal impact on bioenergetics. Cell Rep. 2019;85:1557-1572.e8. 10.1016/j.celrep.2019.01.057. 10.1016/j.celrep.2019.01.057 PMC6478502 30726738

[84] Peoples JN, Ghazal N, Duong DM, Hardin KR, Manning JR, Seyfried NT, et al. Loss of the mitochondrial phosphate carrier SLC25A3 induces remodeling of the cardiac mitochondrial protein acylome. Am J Physiol Cell Physiol. 2021;86:C519–34. 10.1152/ajpcell.00156.2021. 10.1152/ajpcell.00156.2021 PMC8461815 34319827

[85] Qian J, Fulton D. Post-translational regulation of endothelial nitric oxide synthase in vascular endothelium. Front Physiol. 2013. 10.3389/fphys.2013.00347. 24379783 10.3389/fphys.2013.00347 PMC3861784

[86] Yu B-B, Zhi H, Zhang X-Y, Liang J-W, He J, Su C, et al. Mitochondrial dysfunction-mediated decline in angiogenic capacity of endothelial progenitor cells is associated with capillary rarefaction in patients with hypertension via downregulation of CXCR4/JAK2/SIRT5 signaling. EBioMedicine. 2019;42:64–75. 10.1016/j.ebiom.2019.03.031. 30904607 10.1016/j.ebiom.2019.03.031 PMC6491423

[87] Pan Y, Zhang Q, Li C, Li X, Li S, Wang Y, et al. SIRT5 alleviates apoptosis of vascular endothelial cells under simulated microgravity via desuccinylation of ERO1A. Int J Mol Sci. 2025;89:2908. 10.3390/ijms26072908. 10.3390/ijms26072908 PMC11988372 40243486

[88] Zhang H, Zhang Y, Wang H, Yang P, Lu C, Liu Y, et al. Global proteomic analysis reveals lysine succinylation contributes to the pathogenesis of aortic aneurysm and dissection. J Proteomics. 2023;90:104889. 10.1016/j.jprot.2023.104889. 10.1016/j.jprot.2023.104889 36966968

[89] Zhang Y, Zhang H, Wang H, Wang C, Yang P, Lu C, et al. Tandem mass tag-based quantitative proteomic analysis identification of succinylation related proteins in pathogenesis of thoracic aortic aneurysm and aortic dissection. PeerJ. 2023;11:e15258. 10.7717/peerj.15258. 37193023 10.7717/peerj.15258 PMC10183161

[90] Chouchani ET, Pell VR, Gaude E, Aksentijević D, Sundier SY, Robb EL, et al. Ischaemic accumulation of succinate controls reperfusion injury through mitochondrial ROS. Nature. 2014;515:431–5. 10.1038/nature13909. 25383517 10.1038/nature13909 PMC4255242

[91] Liu L, Wang Q, Zhao B, Wu Q, Wang P. Exogenous nicotinamide adenine dinucleotide administration alleviates ischemia/reperfusion-induced oxidative injury in isolated rat hearts via Sirt5-SDH-succinate pathway. Eur J Pharmacol. 2019;858:172520. 10.1016/j.ejphar.2019.172520. 31278893 10.1016/j.ejphar.2019.172520

[92] Boylston JA, Sun J, Chen Y, Gucek M, Sack MN, Murphy E. Characterization of the cardiac succinylome and its role in ischemia–reperfusion injury. J Mol Cell Cardiol. 2015;88:73–81. 10.1016/j.yjmcc.2015.09.005. 26388266 10.1016/j.yjmcc.2015.09.005 PMC4780049

[93] Ali HR, Michel CR, Lin YH, McKinsey TA, Jeong MY, Ambardekar AV, et al. Defining decreased protein succinylation of failing human cardiac myofibrils in ischemic cardiomyopathy. J Mol Cell Cardiol. 2020;138:304–17. 31836543 10.1016/j.yjmcc.2019.11.159 PMC7058372

[94] Zhou B, Xiao M, Hu H, Pei X, Xue Y, Miao G, et al. Cardioprotective role of SIRT5 in response to acute ischemia through a novel liver-cardiac crosstalk mechanism. Front Cell Dev Biol. 2021;9:687559. 34368135 10.3389/fcell.2021.687559 PMC8339556

[95] Ou T, Yang W, Li W, Lu Y, Dong Z, Zhu H, et al. <article-title update="added">SIRT5 deficiency enhances the proliferative and therapeutic capacities of adipose‐derived mesenchymal stem cells via metabolic switching. Clin Transl Med. 2020;10(5):e172. 10.1002/ctm2.172. 32997407 10.1002/ctm2.172 PMC7510333

[96] Li S, Shen S, Hong Y, Ding K, Chen S, Chen J, Wang C, Wen Y, Mo G, Yu L, et al. Sirt5 preserves cardiac function in ischemia-reperfusion injury by inhibiting ANT2 lactylation 2024. 10.1101/2024.11.25.6215148.

[97] Chang X, Zhang Q, Huang Y, Liu J, Wang Y, Guan X, et al. <article-title update="added"> Quercetin inhibits necroptosis in cardiomyocytes after ischemia–reperfusion via <scp>DNA‐PKcs‐SIRT5</scp> ‐orchestrated mitochondrial quality control. Phytother Res. 2024;38(5):2496–517. 10.1002/ptr.8177. 38447978 10.1002/ptr.8177

[98] Wang Y, Zhou H, Wu J, Ye S. MG53 alleviates hypoxia/reoxygenation-induced cardiomyocyte injury by succinylation and ubiquitination modification. Clin Exp Hypertens. 2023;45:2271196. 10.1080/10641963.2023.2271196. 37848382 10.1080/10641963.2023.2271196

[99] Opacic D, van Bragt KA, Nasrallah HM, Schotten U, Verheule S. Atrial metabolism and tissue perfusion as determinants of electrical and structural remodelling in atrial fibrillation. Cardiovasc Res. 2016;109:527–41. 26786160 10.1093/cvr/cvw007

[100] Tu T, Zhou S, Liu Z, Li X, Liu Q. Quantitative proteomics of changes in energy metabolism-related proteins in atrial tissue from valvular disease patients with permanent atrial fibrillation. Circ J. 2014;78:993–1001. 10.1253/circj.CJ-13-1365. 24553264 10.1253/circj.cj-13-1365

[101] Ozcan C, Li Z, Kim G, Jeevanandam V, Uriel N. Molecular mechanism of the association between atrial fibrillation and heart failure includes energy metabolic dysregulation due to mitochondrial dysfunction. J Card Fail. 2019;25:911–20. 10.1016/j.cardfail.2019.08.005. 31415862 10.1016/j.cardfail.2019.08.005 PMC7144800

[102] Bai F, Tu T, Qin F, Ma Y, Liu N, Liu Y, et al. Quantitative proteomics of changes in succinylated proteins expression profiling in left appendages tissue from valvular heart disease patients with atrial fibrillation. Clin Chim Acta. 2019;495:345–54. 10.1016/j.cca.2019.05.002. 31059701 10.1016/j.cca.2019.05.002

[103] Zhang Y, Gong H, Jin L, Liu P, Fan J, Qin X, et al. Succinate predisposes mice to atrial fibrillation by impairing mitochondrial function via SUCNR1/AMPK axis. Redox Biol. 2025;81:103576. 10.1016/j.redox.2025.103576. 40031129 10.1016/j.redox.2025.103576 PMC11915173

[104] Nelson SE, Ament Z, Wolcott Z, Gerszten RE, Kimberly WT. Succinate links atrial dysfunction and cardioembolic stroke. Neurology. 2019;92(8):e802–10. 10.1212/WNL.0000000000006957. 30674589 10.1212/WNL.0000000000006957 PMC6396969

[105] Hu C-K, Huang W-Z, He L, Chang C, Ren Y-L, Dai R-X, et al. De-succinylation-induced accumulation of TRMT10C in the nucleus plays a detrimental role in coronary microembolization via its m1A modification function. Int J Biol Sci. 2025;21(7):2891–920. 10.7150/ijbs.107965. 40384859 10.7150/ijbs.107965 PMC12080399

[106] Liberale L, Akhmedov A, Vlachogiannis NI, Bonetti NR, Nageswaran V, Miranda MX, et al. Sirtuin 5 promotes arterial thrombosis by blunting the fibrinolytic system. Cardiovasc Res. 2021;117(10):2275–88. 10.1093/cvr/cvaa268. 32931562 10.1093/cvr/cvaa268

[107] Zhang L, Lv T, Hou P, Jin Y, Jia F. Sirt5-mediated polarization and metabolic reprogramming of macrophage sustain brain function following ischemic stroke. Brain Res. 2025;1857:149613. 10.1016/j.brainres.2025.149613. 40180144 10.1016/j.brainres.2025.149613

[108] Xiao Z-P, Lv T, Hou P-P, Manaenko A, Liu Y, Jin Y, et al. Sirtuin 5-mediated lysine desuccinylation protects mitochondrial metabolism following subarachnoid hemorrhage in mice. Stroke. 2021;52:4043–53. 10.1161/STROKEAHA.121.034850. 34807744 10.1161/STROKEAHA.121.034850

[109] Xia Q, Zhang X, Zhan G, Zheng L, Mao M, Zhao Y, et al. A cell-penetrating peptide exerts therapeutic effects against ischemic stroke by mediating the lysosomal degradation of sirtuin 5. MedComm. 2023;4:e436. 10.1002/mco2.436. 38093788 10.1002/mco2.436 PMC10716672

[110] Xia Q, Yu Y, Zhan G, Zhang X, Gao S, Han T, et al. The sirtuin 5 inhibitor MC3482 ameliorates microglia‑induced neuroinflammation following ischaemic stroke by upregulating the succinylation level of annexin-A1. J Neuroimmune Pharmacol. 2024;19(1):17. 10.1007/s11481-024-10117-x. 38717643 10.1007/s11481-024-10117-x

[111] Xia Q, Gao S, Han T, Mao M, Zhan G, Wang Y, et al. Sirtuin 5 aggravates microglia-induced neuroinflammation following ischaemic stroke by modulating the desuccinylation of annexin-A1. J Neuroinflammation. 2022;19:301. 10.1186/s12974-022-02665-x. 36517900 10.1186/s12974-022-02665-x PMC9753274

[112] Diaz-Cañestro C, Merlini M, Bonetti NR, Liberale L, Wüst P, Briand-Schumacher S, et al. Sirtuin 5 as a novel target to blunt blood–brain barrier damage induced by cerebral ischemia/reperfusion injury. Int J Cardiol. 2018;260:148–55. 10.1016/j.ijcard.2017.12.060. 29622432 10.1016/j.ijcard.2017.12.060

[113] Li J, Wei G, Song Z, Chen Z, Gu J, Zhang L, et al. SIRT5 regulates ferroptosis through the Nrf2/HO-1 signaling axis to participate in ischemia-reperfusion injury in ischemic stroke. Neurochem Res. 2024;49:998–1007. 10.1007/s11064-023-04095-4. 38170384 10.1007/s11064-023-04095-4

[114] Caturano A, Vetrano E, Galiero R, Salvatore T, Docimo G, Epifani R, et al. Cardiac hypertrophy: from pathophysiological mechanisms to heart failure development. Rev Cardiovasc Med. 2022;23:165. 39077592 10.31083/j.rcm2305165 PMC11273913

[115] Sunagawa Y, Funamoto M, Hamabe-Horiike T, Hieda K, Yabuki S, Tomino M, Ikai Y, Suzuki A, Ogawahara S, Yabuta A, et al. Nobiletin, a polymethoxyflavonoid, activates the desuccinylase activity of SIRT5 and prevents the development of heart failure 2024. 10.1101/2024.01.16.575602.

[116] Zhang N, Zhou Z-Y, Meng Y-Y, Liao H-H, Mou S-Q, Lin Z, et al. <article-title update="added"><scp>HINT2</scp> protects against pressure overload‐induced cardiac remodelling through mitochondrial pathways. J Cell Mol Med. 2024;28:e18276. 10.1111/jcmm.18276. 38546629 10.1111/jcmm.18276 PMC10977391

[117] Liang L, Huang Y, Wang Q, Hong Y, Zhen H, Chen Y. SIRT5 prevents mitochondrial dysfunction and cardiac hypertrophy induced by RIP140. Iran J Basic Med Sci. 2024. 10.22038/ijbms.2024.80343.17390. 39968093 10.22038/ijbms.2024.80343.17390 PMC11831744

[118] Hershberger KA, Abraham DM, Liu J, Locasale JW, Grimsrud PA, Hirschey MD. Ablation of sirtuin5 in the postnatal mouse heart results in protein succinylation and normal survival in response to chronic pressure overload. J Biol Chem. 2018;293:10630–45. 29769314 10.1074/jbc.RA118.002187 PMC6036188

[119] Guo AH, Baliira R, Skinner ME, Kumar S, Andren A, Zhang L, et al. Sirtuin 5 levels are limiting in preserving cardiac function and suppressing fibrosis in response to pressure overload. Sci Rep. 2022;12:12258. 10.1038/s41598-022-16506-7. 35851833 10.1038/s41598-022-16506-7 PMC9293976

[120] Wu L-F, Wang D-P, Shen J, Gao L-J, Zhou Y, Liu Q-H, et al. Global profiling of protein lysine malonylation in mouse cardiac hypertrophy. J Proteomics. 2022;266:104667. 10.1016/j.jprot.2022.104667. 35788409 10.1016/j.jprot.2022.104667

[121] Ritterhoff J, Tian R. Metabolic mechanisms in physiological and pathological cardiac hypertrophy: new paradigms and challenges. Nat Rev Cardiol. 2023;20:812–29. 37237146 10.1038/s41569-023-00887-x

[122] Chen X, Wu H, Liu Y, Liu L, Houser SR, Wang WE. Metabolic reprogramming: a byproduct or a driver of cardiomyocyte proliferation? Circulation. 2024;149:1598–610. 10.1161/CIRCULATIONAHA.123.065880. 38739695 10.1161/CIRCULATIONAHA.123.065880

[123] Wu M, Zhang H, Yuan W, Tan J, Huang Z, Chen Z, et al. Abstract 9328: Sirt5 deficiency promotes metabolic reprogramming to aggravate cardiac fibrosis. Circulation. 2022;146(Suppl_1):A9328–A9328. 10.1161/circ.146.suppl_1.9328.

[124] Chen L, Wang H, Gao F, Zhang J, Zhang Y, Ma R, et al. Functional genetic variants in the SIRT5 gene promoter in acute myocardial infarction. Gene. 2018;675:233–9. 29981421 10.1016/j.gene.2018.07.010

[125] Zhou B, Du Y, Xue Y, Miao G, Wei T, Zhang P. Identification of malonylation, succinylation, and glutarylation in serum proteins of acute myocardial infarction patients. Proteomics Clin Appl. 2020;14:1900103. 10.1002/prca.201900103. 10.1002/prca.201900103 31532912

[126] Li Z, Zheng Z, Dai X. SIRT5 induces autophagy and alleviates myocardial infarction via desuccinylation of TOM1. BMC Cardiovasc Disord. 2024;24:464. 10.1186/s12872-024-04120-6. 39210272 10.1186/s12872-024-04120-6 PMC11363360

[127] Zheng Z, Liu Y, Chen D, Yang J, Ren L, Jin Z, et al. Catalpol improved energy metabolism and inflammation through the SIRT5-mediated signaling pathway to ameliorate myocardial injury. Sci Rep. 2024;14:29240. 39587219 10.1038/s41598-024-80505-z PMC11589681

[128] Chang X, Zhou S, Huang Y, Liu J, Wang Y, Guan X, et al. Zishen huoxue decoction (ZSHX) alleviates ischemic myocardial injury (MI) via Sirt5-β-tubulin mediated synergistic mechanism of “mitophagy-unfolded protein response” and mitophagy. Chin J Nat Med. 2025;23(3):311–21. 10.1016/S1875-5364(25)60838-7. 40122661 10.1016/S1875-5364(25)60838-7

[129] Castiello C, Efentakis P, Nikolaou P-E, Symeonidi L, Chania C, Barla I, et al. Cardioprotection through pharmacological activation of sirtuin 5 in a murine model of acute myocardial infarction. Drug Des Devel Ther. 2025;19:5489–505. 10.2147/DDDT.S509337. 40606000 10.2147/DDDT.S509337 PMC12214431

[130] Sacks D, Baxter B, Campbell BCV, Carpenter JS, Cognard C, Dippel D, Eesa M, Fischer U, Hausegger K, et al.; From the American Association of Neurological Surgeons (AANS), American Society of Neuroradiology (ASNR), Cardiovascular and Interventional Radiology Society of Europe (CIRSE), Canadian Interventional Radiology Association (CIRA), Congress of Neurological Surgeons (CNS), European Society of Minimally Invasive Neurological Therapy (ESMINT), European Society of Neuroradiology (ESNR), European Stroke Organization (ESO), Society for Cardiovascular Angiography and Interventions (SCAI), Society of Interventional Radiology (SIR), Society of NeuroInterventional Surgery (SNIS), and World Stroke Organization (WSO). Multisociety consensus quality improvement revised consensus statement for endovascular therapy of acute ischemic stroke. Int J Stroke. 2018;13:612–632. 10.1177/1747493018778713.

[131] Tang L, Sun Q, Luo J, Peng S. Metformin hydrochloride improves hepatic glucolipid metabolism in diabetes progression through SIRT5-mediated ECHA desuccinylation. Sci Rep. 2025;15:7768. 10.1038/s41598-025-92716-z. 40044936 10.1038/s41598-025-92716-z PMC11882834

[132] Bagul PK, Dinda AK, Banerjee SK. Effect of resveratrol on sirtuins expression and cardiac complications in diabetes. Biochem Biophys Res Commun. 2015;468:221–7. 10.1016/j.bbrc.2015.10.126. 26518647 10.1016/j.bbrc.2015.10.126

[133] Wei C, Shi M, Dong S, Li Z, Zhao B, Liu D, et al. SIRT5-related lysine demalonylation of GSTP1 contributes to cardiomyocyte pyroptosis suppression in diabetic cardiomyopathy. Int J Biol Sci. 2024;20:585–605. 10.7150/ijbs.83306. 38169591 10.7150/ijbs.83306 PMC10758093

[134] Wu M, Tan J, Cao Z, Cai Y, Huang Z, Chen Z, et al. Sirt5 improves cardiomyocytes fatty acid metabolism and ameliorates cardiac lipotoxicity in diabetic cardiomyopathy via CPT2 de-succinylation. Redox Biol. 2024;73:103184. 38718533 10.1016/j.redox.2024.103184 PMC11091707

[135] Rosca MG, Hoppel CL. Mitochondrial dysfunction in heart failure. Heart Fail Rev. 2013;18:607–22. 22948484 10.1007/s10741-012-9340-0 PMC3855291

[136] Rosca MG, Hoppel CL. Mitochondria in heart failure. Cardiovasc Res. 2010;88:40–50. 20668004 10.1093/cvr/cvq240 PMC3025720

[137] Marín-García J, Akhmedov AT, Moe GW. Mitochondria in heart failure: the emerging role of mitochondrial dynamics. Heart Fail Rev. 2013;18:439–56. 22707247 10.1007/s10741-012-9330-2

[138] Takada S, Maekawa S, Furihata T, Kakutani N, Setoyama D, Ueda K, et al. Succinyl-CoA-based energy metabolism dysfunction in chronic heart failure. Proc Natl Acad Sci U S A. 2022;119:e2203628119. 10.1073/pnas.2203628119. 36201541 10.1073/pnas.2203628119 PMC9564216

[139] Jeremy Jenkins, PhD | BioRender Available online: https://app.biorender.com/profile/template/details/t-67f53c29e8ca693ea663656d-target-identification-in-ai-drug-discovery/?source=profile&username=jeremyjenkins&tab=templates Accessed 19 Oct 2025.

[140] Spray L, Richardson G, Haendeler J, Altschmied J, Rumampouw V, Wallis SB, et al. Cardiovascular inflammaging: mechanisms, consequences, and therapeutic perspectives. Cell Rep Med. 2025. 10.1016/j.xcrm.2025.102264. 40782796 10.1016/j.xcrm.2025.102264 PMC12490256

